# Adiponectin, Leptin, and IGF-1 Are Useful Diagnostic and Stratification Biomarkers of NAFLD

**DOI:** 10.3389/fmed.2021.683250

**Published:** 2021-06-23

**Authors:** Vanda Marques, Marta B. Afonso, Nina Bierig, Filipa Duarte-Ramos, Álvaro Santos-Laso, Raul Jimenez-Agüero, Emma Eizaguirre, Luis Bujanda, Maria J. Pareja, Rita Luís, Adília Costa, Mariana V. Machado, Cristina Alonso, Enara Arretxe, José M. Alustiza, Marcin Krawczyk, Frank Lammert, Dina G. Tiniakos, Bertram Flehmig, Helena Cortez-Pinto, Jesus M. Banales, Rui E. Castro, Andrea Normann, Cecília M. P. Rodrigues

**Affiliations:** ^1^Research Institute for Medicines (iMed.ULisboa), Faculty of Pharmacy, Universidade de Lisboa, Lisbon, Portugal; ^2^Mediagnost, GmbH, Reutlingen, Germany; ^3^EPIUnit–Instituto de Saúde Pública, Universidade do Porto, Oporto, Portugal; ^4^Department of Liver and Gastrointestinal Diseases, Biodonostia Health Research Institute, Donostia University Hospital, University of the Basque Country (UPV/EHU), San Sebastian, Spain; ^5^National Institute for the Study of Liver and Gastrointestinal Diseases (CIBERehd, Instituto de Salud Carlos III), Madrid, Spain; ^6^Hospital de Valme, Sevilla, Spain; ^7^Department of Pathological Anatomy, Hospital de Santa Maria, Centro Hospitalar Universitário Lisboa Norte, Lisbon, Portugal; ^8^Faculdade de Medicina, Clinica Universitária de Gastrenterologia, Universidade de Lisboa, Lisbon, Portugal; ^9^Department of Gastroenterology, Hospital de Santa Maria, Centro Hospitalar Universitário Lisboa Norte, Lisbon, Portugal; ^10^OWL Metabolomics, Bizkaia Technology Park, Derio, Spain; ^11^Radiology Service, Osatek, Donostia, Spain; ^12^Department of Medicine II, Saarland University Medical Center, Homburg, Germany; ^13^Laboratory of Metabolic Liver Diseases, Department of General, Transplant and Liver Surgery, Centre for Preclinical Research, Medical University of Warsaw, Warsaw, Poland; ^14^Faculty of Medical Sciences, Translational and Clinical Research Institute, Newcastle University, Newcastle upon Tyne, United Kingdom; ^15^Department of Pathology, Aretaieio Hospital, National and Kapodistrian University of Athens, Athens, Greece; ^16^IKERBASQUE, Basque Foundation for Science, Bilbao, Spain

**Keywords:** adiponectin, circulating biomarkers, fibrosis, IGF-1, leptin, lipid metabolism, NAFLD

## Abstract

**Background:** Nonalcoholic fatty liver disease (NAFLD) is the most common chronic liver disease where liver biopsy remains the gold standard for diagnosis. Here we aimed to evaluate the role of circulating adiponectin, leptin, and insulin-like growth factor 1 (IGF-1) levels as non-invasive NAFLD biomarkers and assess their correlation with the metabolome.

**Materials and Methods:** Leptin, adiponectin, and IGF-1 serum levels were measured by ELISA in two independent cohorts of biopsy-proven obese NAFLD patients and healthy-liver controls (discovery: 38 NAFLD, 13 controls; validation: 194 NAFLD, 31 controls) and correlated with clinical data, histology, genetic parameters, and serum metabolomics.

**Results:** In both cohorts, leptin increased in NAFLD vs. controls (discovery: AUROC 0.88; validation: AUROC 0.83; *p* < 0.0001). The leptin levels were similar between obese and non-obese healthy controls, suggesting that obesity is not a confounding factor. In the discovery cohort, adiponectin was lower in non-alcoholic steatohepatitis (NASH) vs. non-alcoholic fatty liver (AUROC 0.87; *p* < 0.0001). For the validation cohort, significance was attained for homozygous for PNPLA3 allele c.444C (AUROC 0.63; *p* < 0.05). Combining adiponectin with specific serum lipids improved the assay performance (AUROC 0.80; *p* < 0.0001). For the validation cohort, IGF-1 was lower with advanced fibrosis (AUROC 0.67, *p* < 0.05), but combination with international normalized ratio (INR) and ferritin increased the assay performance (AUROC 0.81; *p* < 0.01).

**Conclusion:** Serum leptin discriminates NAFLD, and adiponectin combined with specific lipids stratifies NASH. IGF-1, INR, and ferritin distinguish advanced fibrosis.

## Introduction

Non-alcoholic fatty liver disease (NAFLD) is one of the most prevalent chronic liver conditions and an important risk factor for liver cirrhosis and hepatocellular carcinoma. The NAFLD spectrum varies from simple fat accumulation to non-alcoholic steatohepatitis (NASH), characterized by hepatocellular injury, inflammation, and, eventually, fibrosis (1). In fact, the increasing prevalence of NAFLD parallels that of obesity and is accompanied by rising liver-related morbidity and mortality and, consequently, increased socio-economic burden (2). Clinical practice guidelines recommend NAFLD screening in individuals with metabolic risk factors, such as obesity, type 2 diabetes, and hypertension (metabolic syndrome) (3, 4). Most NAFLD patients are clinically asymptomatic, and when present, the symptoms are unspecific and associated with advanced disease. Furthermore, liver function tests may be within normal range or only slightly increased. Thus, panels composed by anthropometric and blood biochemistry data have been proposed to assess hepatic steatosis (fatty liver index, hepatic steatotic index, NAFLD liver fat score, SteatoTest), although with modest performance compared with imaging biomarkers. In turn, imaging techniques [ultrasonography, magnetic resonance imaging (MRI)] are not fully conclusive at identifying liver inflammation and hepatocyte injury typical of NASH or distinguishing NASH from fatty liver (3, 4). Indeed differential diagnosis between non-alcoholic fatty liver (NAFL) and NASH is an important indicator of increased risk of cirrhosis and other hepatic co-morbidities, although the stage of fibrosis is increasingly recognized as the most relevant NASH prognostic marker (5).

Caspase-3-generated cytokeratin-18 is the most studied biomarker for NASH diagnosis but has limited sensitivity for NASH stage screening (6); hence, other putative biomarkers (inflammatory markers, adipokines, and others) are emerging (7). In parallel, biomarker panels combining several blood parameters are being proposed for fibrosis staging, such as aspartate aminotransferase (AST) to alanine aminotransferase (ALT) ratio, AST/platelet ratio index (APRI), fibrosis-4 index (Fib-4), or FibroTest, but accuracy has been limited as they mostly rely on liver injury markers (8). To date, liver biopsy is the gold-standard method to accurately discriminate NAFL from NASH, to evaluate the extent of tissue damage and fibrosis, and to rule out other etiologies. However, this is an expensive and invasive procedure with potential for serious complications (9). Moreover, liver biopsy lacks the ability to provide a complete 3D liver overview and is limited by sampling variability. Hence, identification and validation of simple, specific, reproducible, and non-invasive biomarkers that accurately diagnose and monitor NAFLD patients constitute an urgent unmet medical need. NAFLD is generally regarded as a hepatic manifestation of metabolic syndrome (10). Although not yet fully understood, the disease pathogenesis involves disturbances in adipose tissue, insulin resistance, and inflammation (11). Thus, adipokines' and related cytokines' role in NAFLD pathogenesis and severity has been equated (12), and their potential as non-invasive biomarkers of diagnosis and staging deserves further investigation. Moreover, lipid and metabolite signatures in plasma and in liver tissue associated with NAFLD and/or fibrosis (13–15) may further support non-invasive NAFLD diagnosis (16).

We hypothesize that serum adipokines—leptin and adiponectin—and liver-produced insulin-like growth factor 1 (IGF-1) are useful circulating biomarkers for NAFLD diagnosis and stratification. We assessed their potential as non-invasive biomarkers in the discovery and validation cohorts of biopsy-proven NAFLD patients and evaluated their impact on the metabolome.

## Materials and Methods

### Patient Cohorts

Human serum samples were collected from two independent cohorts of morbidly obese adult patients with biopsy-proven diagnosis of NAFLD, who were undergoing bariatric surgery [body mass index (BMI) ≥35 kg/m^2^], and healthy-liver individuals (17) (BMI <35 kg/m^2^) from Santa Maria Hospital (Lisbon, Portugal; discovery cohort) and Donostia University Hospital (San Sebastian, Spain; validation cohort). Up to 20% of the patients were taking statins. The patient inclusion criteria consisted in liver biopsy-proven NAFLD diagnosis and BMI ≥35 kg/m^2^. The exclusion criteria included alcohol consumption ≥20 (females) or ≥30 g/day (males), viral hepatitis B and C, and other causes of chronic liver disease. The liver biopsies were obtained during bariatric surgery, under standard procedures. Fibrosis was assessed from a distance >0.5 cm from the liver capsule. Blood and tissue samples were collected at a maximum of 1-week interval, while liver imaging was performed within 24 h of surgery. Sample collection was performed after receipt of the patient's informed consent and the Institutional Review Board's approval, in accordance with the Declaration of Helsinki. For histology purposes, formalin-fixed paraffin-embedded tissue sections were routinely stained with hematoxylin–eosin method and with Masson's trichrome for fibrosis evaluation. Histological evaluation was performed by experienced pathologists in a blinded fashion following agreement on the histological features of NAFLD/NASH assessment and NASH diagnosis. NAFLD activity score (NAS 0–8) and fibrosis stage (F0–4) were assigned to each biopsy according to the NASH Clinical Research Network histological scoring system (18). Fibrosis extent was dichotomized as early (F0–2) and advanced (F3–4) fibrosis. Where possible, fibrosis scores APRI (19), Fib-4 (20), and NAFLD fibrosis score (NFS) (21) were calculated. Individuals were classified as healthy non-obese controls (BMI <35 kg/m^2^), healthy obese controls [BMI ≥35 kg/m^2^, maximum NAS = 1 (if steatosis = 0) and maximum fibrosis score = 1], NAFL (NAS ≤ 4), or NASH (NAS ≥5). Liver biopsies were obtained from healthy non-obese individuals that were referred for hepatic surgery without an underlying liver disease (17).

The discovery cohort (*n* = 51) included three groups: healthy non-obese controls (*n* = 13), NAFL (*n* = 26), and NASH patients (*n* = 12). Routine biochemical parameters were available; however, detailed histological data was not fully disclosed.

The validation cohort (*n* = 225) encompassed four groups: healthy non-obese controls (*n* = 20), healthy obese controls (*n* = 11), NAFL (*n* = 100), and NASH patients (*n* = 94). Routine biochemical parameters were available, and detailed histological data is described in Supplementary Table 1. Information on co-morbidities (arterial hypertension, diabetes, dyslipidemia, and cholelithiasis), genetic polymorphisms [*PNPLA3* rs738409 (c.444C>G, p.I148M), *MBOAT7* rs641738 (c.50G>A, p.G17E), and *TM6SF2* rs58542926 (c.449C>T, p.E167K)], liver hepatic triglyceride (TG) content evaluation by Folch method (17) (*n* = 140), and imaging assessment of liver steatosis (MRI; *n* = 122) were also available.

### Serum Hormones and Data Analysis

The serum levels of leptin, adiponectin, and IGF-1 were measured in single determination using specific enzyme-linked immunosorbent assay kits according to the manufacturer's instructions (Mediagnost GmbH, Reutlingen, Germany) and correlated with NAFLD activity score, fibrosis stage, biochemical parameters, genotypes, imaging data, and co-morbidities. Data analysis was first performed in the discovery cohort and then expanded to the validation cohort. The normality of values distribution was tested with Kolmogorov–Smirnov (*n* ≥ 50) or Shapiro–Wilk (*n* < 50) tests. According to the normality of values distribution, one-way ANOVA or Kruskal–Wallis test followed by Bonferroni or Dunn's multiple-comparison test were used to determine differences between sample groups according to NAS score, steatosis, lobular inflammation and ballooning stages, and genotypes. Differences according to fibrosis dichotomy and presence of co-morbidities were determined with unpaired *t*-test or Mann–Whitney *U*-test. To correlate hormone serum levels and biochemical parameters or imaging data, Pearson or Spearman correlation coefficient and linear regression analysis were applied. Uniparameter receiver operating characteristic (ROC) curve analysis tested the hormones' discriminatory power. Cutoff values were retrieved from Youden index. Variables presenting an area under the ROC curve (AUROC) value superior to 0.60 and *p* < 0.05 were selected for binomial logistic regression analysis. Multi-parameter ROC curves were established for relevant parameter combinations, where an AUROC value >0.80 was considered a very good assay performance. Associated sensitivity, specificity, and positive predictive value (PPV) and negative predictive value (NPV) were estimated. A *p*-value <0.05 was considered significant. All statistical analysis were performed using GraphPad Prism, version 8.0.2 (La Jolla, California, USA) and IBM SPSS Statistics, version 26 (Armonk, New York, USA).

### Metabolomic Analysis

Serum samples from 198 patients in the validation cohort were randomly selected for metabolomic analysis by liquid chromatography–time of flight–mass spectrometry. Two separate liquid chromatography–time of flight–mass spectrometry-based platforms were used: platform 1 to analyze methanol lipid extracts [including fatty acids (FA), acyl carnitines, bile acids, lysoglycerophospholipids, N-acyl ethanolamines, and oxidized FA] and platform 2 for methanol/chloroform lipid extracts (including glycerolipids, cholesterol esters, sphingolipids, and glycerophospholipids) (22). Quality control calibration and validation samples were used to assess data quality. The spectra were mass-corrected by reference to leucine enkephalin. System control and data pre-processing were performed using Masslynx 4.1 software and TargetLynx application manager (Waters Corp., Milford, USA). Peak detection, noise reduction, and data normalization were performed as previously described (23). For univariate analyses, the samples were grouped according to the median serum level of each hormone (leptin = 39.61 ng/ml; adiponectin = 4.84 μg/ml; IGF-1 = 103.65 ng/ml). Log_2_ fold change for metabolite levels between higher than median vs. lower than median hormone level groups was determined and compared using unpaired Student's *t*-test (*p* < 0.05) using R v.3.4.1 (R Development Core Team, 2017; http://cran.r-project.org). A *p*-value <0.05 was considered significant.

## Results

### Clinical and Biochemical Features of Patient Cohorts

In both cohorts, the NAFL and NASH patients were similar with respecting to age, gender, BMI, gamma-glutamyl transferase (GGT), serum glucose, triglycerides, and cholesterol [total, high-density lipoprotein (HDL) or low-density lipoprotein (LDL)] concentrations (Tables 1, 2). While in the discovery cohort the NAFL and NASH patients had similar circulating AST and ALT, in the validation cohort these liver injury markers and liver fat content evaluated by magnetic resonance imaging and Folch method were increased in NASH vs. NAFL (*p* < 0.05). The prevalence of co-morbidities was similar between NAFL and NASH patients, except for dyslipidemia (higher in NASH vs. NAFL; *p* < 0.05). *PNPLA3* risk allele c.444G frequency vs. wild-type allele was lower in both controls (12.5 vs. 87.5%) and NAFLD (23.7 vs. 76.3%), consistent with previous reports (24). Similarly to previous literature (25), in NAFLD patients, *TM6SF2* risk allele c.499T showed a frequency of 5.7%, while *MBOAT7* risk allele c.50>A presented a frequency of 45.7%.

**Table 1:** Patient demographic and clinical and laboratory data for the discovery cohort.

**Parameter**	**NAFL**	**NASH**
Number	26	12
Age (years)	38.38 ± 1.99	44.83 ± 2.68
Gender (M/F)	10/16	4/8
BMI (kg/m^2^)	44.68 ± 1.32	41.56 ± 1.27
**Hematology and biochemistry**
AST (U/L)	27.19 ± 2.55	30.92 ± 3.16
ALT (U/L)	39.62 ± 6.96	45.08 ± 5.38
GGT (U/L)	29.36 ± 4.22	37.58 ± 3.42
Glucose (mg/dl)	93.58 ± 2.13	100.58 ± 3.68
Triglycerides (mg/ml)	118.44 ± 5.82	155.91 ± 20.63
Total cholesterol (mg/ml)	191.27 ± 7.41	197.67 ± 10.83
HDL-cholesterol (mg/ml)	46.27 ± 2.69	44.08 ± 2.57
LDL-cholesterol (mmol/dl)	125.00 ± 7.61	125.73 ± 10.11
Total bilirubin (mg/dl)	0.69 ± 0.08	0.74 ± 0.08
Platelets (10^9^/L)	280.42 ± 17.32	300.82 ± 11.70
INR	0.97± 0.01	0.94 ± 0.02

*Data are expressed as mean ± SEM. Information not available for non-obese controls*.

*ALT, alanine aminotransferase; AST, aspartate aminotransferase; BMI, body mass index; F, female; GGT, γ-glutamyl transferase; HDL, high-density lipoprotein; INR, international normalized ratio; LDL, low-density lipoprotein; M, male; NAFL, nonalcoholic fatty liver; NASH, nonalcoholic steatohepatitis*.

**Table 2:** Patient demographic, clinical and laboratory data for validation cohort.

**Parameter**	**Obese controls**	**NAFL**	**NASH**
Number	11	100	94
Age (years)	48.10 ± 2.86	45.20 ± 1.27	48.96 ± 1.67
Gender (M/F)	4/6	26/66	34/56
BMI (kg/m^2^)	46.03 ± 1.87	44.60 ± 0.67	45.89 ± 0.73
**Hematology and biochemistry**			
AST (U/L)	23.78 ± 5.93	20.71 ± 0.92	26.24 ± 1.49^a^
ALT (U/L)	22.67 ± 3.43	25.68 ± 1.70	35.18 ± 2.21^a^
GGT (U/L)	21.14 ± 3.52	37.20 ± 4.78	42.27 ± 4.45
Glucose (mg/dl)	108.30 ± 6.77	113.65 ± 3.44	120.22 ± 3.39
Triglycerides (mg/ml)	127.00 ± 32.44	142.22 ± 9.98	158.79 ± 8.43
Total cholesterol (mg/ml)	198.25 ± 4.91	195.75 ± 4.08	206.79 ± 5.41
HDL-cholesterol (mg/ml)	51.50 ± 4.84	48.58 ± 1.47	50.24 ± 1.45
LDL-cholesterol (mmol/dl)	131.30 ± 8.31	118.38± 3.65	125.69 ± 4.12
Total bilirubin (mg/dl)	0.54 ± 0.10	0.55 ± 0.09	0.48 ± 0.03
Platelets (10^9^/L)	281.00 ± 33.57	267.78 ± 7.09	251.79 ± 6.22
Hemoglobin (g/dl)	12.40 ± 0.61	13.73 ± 0.13	13.75 ± 0.21
Ferritin (ng/ml)	166.87 ± 31.14	114.15 ± 15.91	134.34 ± 19.79
Albumin (g/dl)	3.96 ± 0.27	4.28 ± 0.05	4.28 ± 0.06
INR	1.01 ± 0.02	0.97 ± 0.01	0.98 ± 0.01
**Liver steatosis**			
MRI (fat fraction)	n/a	0.15 ± 0.01	0.26 ± 0.01^a^
Hepatic TG content evaluated by Folch method (mg/g)	n/a	57.49 ± 5.47	103.13 ± 6.42^a^
**NAFLD-associated polymorphisms**			
PNPLA3 (CC/CG/GG)	7/0/1	59/24/6	47/28/9
TM6SF2 (CC/CT/TT)	8/0/0	63/5/3	65/5/0
MBOAT7 (GG/GA/AA)	4/4/0	21/50/18	27/42/15
**Co-morbidities**			
Arterial hypertension (no/yes)	6/4	46/45	31/59
Diabetes (no/yes)	5/5	66/26	55/34
Dyslipidemia (no/yes)	3/1	55/23	41/41^a^
Cholelithiasis (no/yes)	3/1	52/23	60/19

*Data are expressed as mean ± SEM. Information not available for non-obese controls*.

*ALT, alanine aminotransferase; AST, aspartate aminotransferase; BMI, body mass index; C, cytosine; F, female; G, guanine; GGT, γ-glutamyl transferase; HDL, high-density lipoprotein; INR, international normalized ratio; LDL, low-density lipoprotein; M, male; MRI, magnetic resonance imaging; n/a, not available; NAFL, nonalcoholic fatty liver; NASH, nonalcoholic steatohepatitis; T, thymine*.

a *p < 0.05 vs. NAFL*.

### Leptin Predicts NAFLD and Correlates With Serum Content

**Figure 1:**
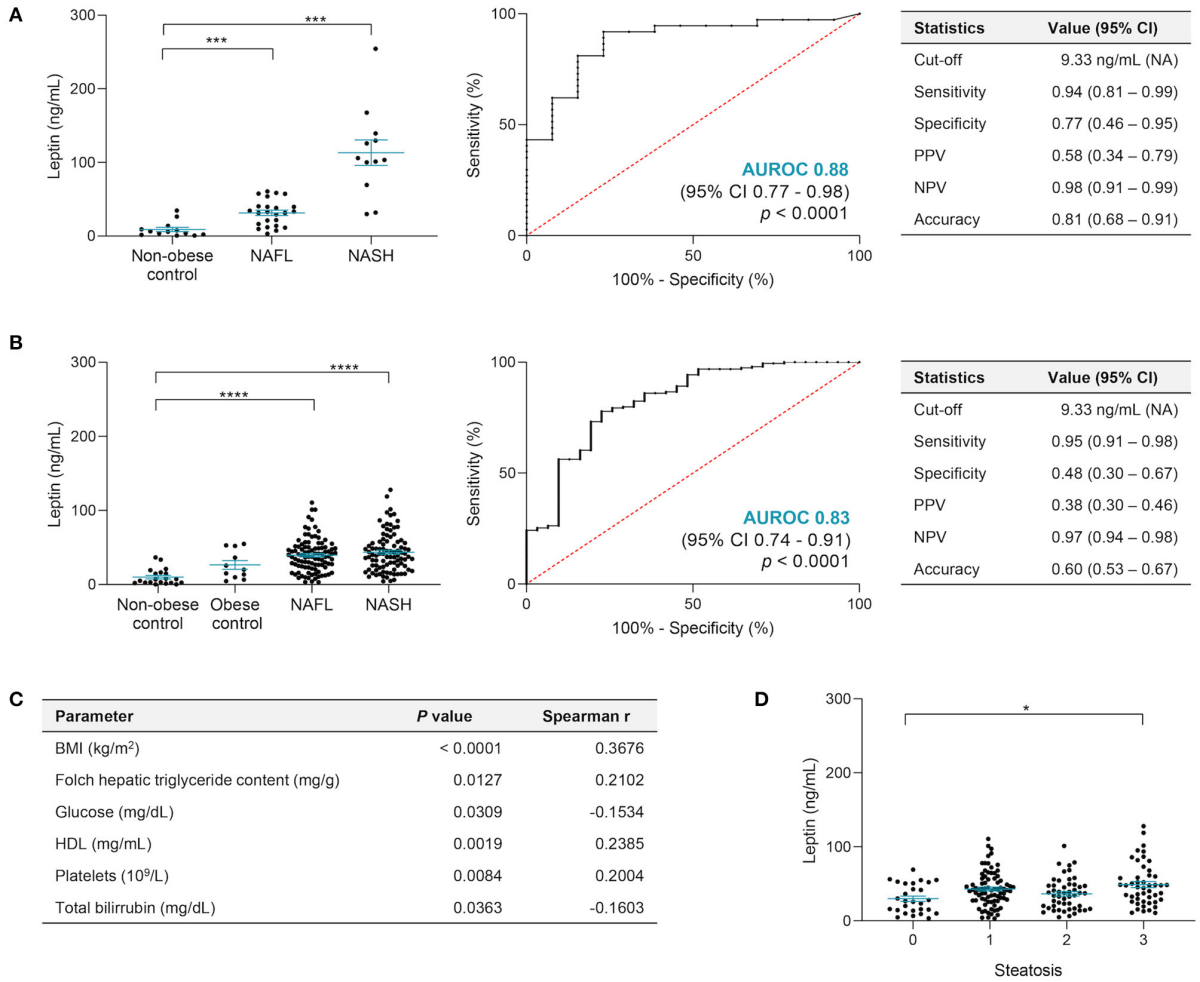
Leptin serum levels allow to distinguish NAFLD. **(A)** In the discovery cohort, leptin levels were significantly higher in NAFL and NASH patients compared with non-obese, healthy-liver controls, presenting an AUROC value of 0.88 when distinguishing controls (*n* = 13) from NAFLD patients (*n* = 36), with very high general accuracy and NPV. **(B)** In the validation cohort, the leptin levels did not show differences among healthy controls, non-obese or obese, and confirmed increased levels in NAFL and NASH. Leptin presented good performance assay results when distinguishing healthy controls (*n* = 31) from NAFLD (*n* = 194), with an AUROC value of 0.83 and overall accuracy of 0.60 when applied the cutoff value of >9.33 ng/ml leptin for NAFLD diagnosis established in the discovery cohort. **(C)** Main correlations found between leptin and other parameters in the validation cohort: BMI values (*n* = 199), hepatic TG evaluated by Folch method (*n* = 140; mg TG/g liver tissue), glucose (*n* = 198), HDL (*n* = 167), platelets (*n* = 172), and total bilirubin (*n* = 171). **(D)** Leptin levels increased with steatosis severity in the validation cohort. The *x*-axis represents the histological grade of steatosis, ranging from 0 to 3. There was a significant difference between grade 0 vs. 3 (*n* = 28, grade 0; *n* = 80, grade 1; *n* = 49, grade 2; *n* = 48, grade 3). Leptin levels depicted as mean ± SEM. BMI, body mass index; CI, confidence interval; HDL, high-density lipoprotein; NAFL, non-alcoholic fatty liver; NAFLD, nonalcoholic fatty liver disease; NASH, nonalcoholic steatohepatitis; NPV, negative predictive value; PPV, positive predictive value. *p < 0.05; ***p < 0.001; ****p < 0.0001.

The impact of BMI on leptin and its influence on liver fibrosis remain controversial (26). We observed lower leptin levels in men than in women (discovery cohort: 20.63 ± 3.57 vs. 40.63 ± 4.00 ng/ml, *p* < 0.01; validation cohort: 25.8 ± 2.03 vs. 49.55 ± 1.99 ng/ml, *p* < 0.0001) as reported before (27). In the discovery cohort, serum leptin was higher in NAFL (31.43 ± 3.59 ng/ml, *p* < 0.01) and NASH (37.62 ± 5.12 ng/ml, *p* < 0.001) vs. in non-obese controls (8.92 ± 2.88 ng/ml), showing a good performance when distinguishing non-obese controls from NAFLD patients, with AUROC = 0.88 (95% CI, 0.77–0.98; *p* < 0.0001) and an overall accuracy of 0.81 (95% CI, 0.68–0.91), considering 25% disease prevalence (Figure 1A). By applying a leptin cutoff value >9.33 ng/ml for NAFLD inclusion, the assay sensitivity and specificity were 94 and 77%, respectively, and the NPV for ruling out NAFLD diagnosis was 98% (Figure 1A). No correlations were found between serum leptin and BMI or any biochemical parameter.

In the validation cohort, leptin was higher again in NAFL (39.74 ± 2.25 ng/ml) and NASH (43.72 ± 2.70 ng/ml) vs. non-obese controls (6.21 ± 0.97 ng/ml; *p* < 0.0001) (Figure 1B). Although a positive correlation was found between serum leptin levels and BMI (*p* < 0.0001) (Figure 1C), no significant differences were evident between non-obese and obese controls (26.52 ± 5.82 ng/ml), suggesting that obesity might not be a confounding factor (Figure 1B). Serum leptin showed a good performance again when distinguishing controls (both non-obese and obese) from NAFLD patients (both NAFL and NASH), with AUROC = 0.83 (95%, CI 0.74–0.91; *p* < 0.0001). Considering 25% disease prevalence and by applying the cutoff value >9.33 ng/ml for NAFLD inclusion determined in the discovery cohort, an overall accuracy of 0.60 (95% CI, 0.53–0.67) was achieved, with sensitivity of 95% and specificity of 48% (Figure 1B). Serum leptin positively correlated with hepatic TG content (*p* < 0.05), HDL (*p* < 0.01), platelets (*p* < 0.01), and total bilirubin (*p* < 0.01) (Figure 1C). Curiously, diabetic NAFLD patients had significantly lower leptin compared with those who were non-diabetic (34.90 ± 2.87 vs. 45.39 ± 2.17 ng/ml; *p* < 0.01; Supplementary Figure 1), supported by the negative correlation between leptin and fasting glucose (*p* < 0.05; Figure 1C). Leptin was increased in patients with severe liver steatosis (stage 0 vs. 3, *p* < 0.05) (Figure 1D) and tended to increase with lobular inflammation and hepatic ballooning, while no significant differences were found with other co-morbidities, fibrosis stage, or NAFLD-associated polymorphisms (Supplementary Figure 1).

**Figure 2:**
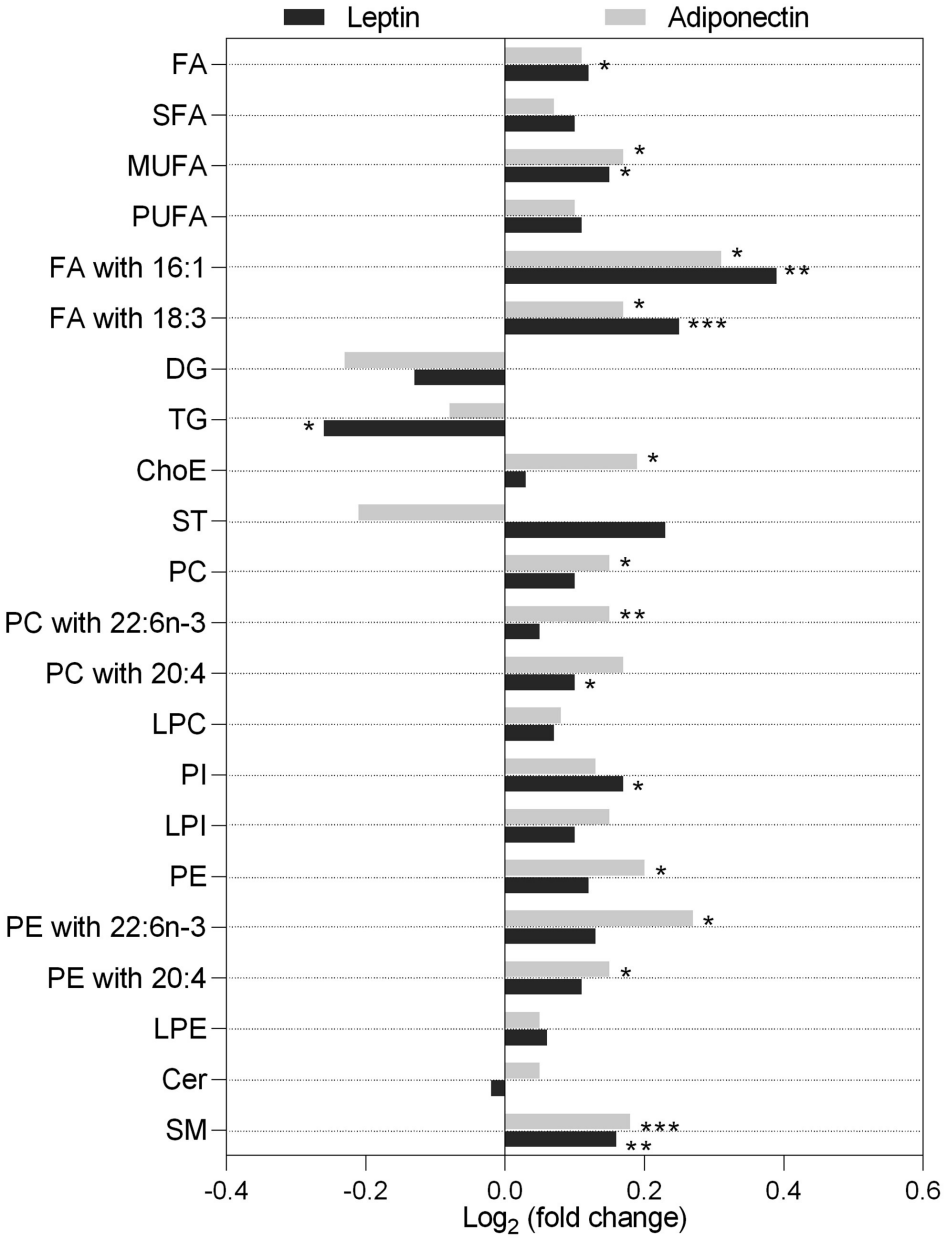
Serum lipidomic signatures associated with higher circulating levels of leptin (dark gray) and adiponectin (light gray). Data presented as log_2_ of the lipid level fold change between patients with hormone levels higher than the median *vs*. lower than the median. Leptin median level = 39.61 ng/ml; higher, *n* = 99; lower, *n* = 99. Adiponectin median level = 4.84 μg/ml; higher, *n* = 95; lower, *n* = 99. Cer, ceramide; ChoE, cholesteryl ester; DG, diglyceride; FA, fatty acid; LPC, lysophosphatidylcholines; LPE, lysophosphatidylethanolamines; LPI, lysophosphatidylinositols; MUFA, monounsaturated FA; PC, phosphatidylcholines; PE, phosphatidylethanolamines; PI, phosphatidylinositols; PUFA, polyunsaturated FA; SFA, saturated FA; SM, sphingomyelins; ST, steroids; TG, triglycerides; 16:0, palmitic acid; 18:3, linolenic acid; 20:4, arachidonic acid; 22:6n-3, docosahexaenoic acid (DHA). *p < 0.05; **p < 0.01; ***p < 0.001.

Given leptin's association with hepatic fat accumulation, its impact on the serum metabolic signatures of NAFLD patients was further evaluated. Levels of circulating lipids and other metabolites in NAFLD patients were compared between high/low levels of circulating leptin. Higher serum leptin was associated with a general increase in serum lipids, namely, FA (*p* < 0.05), monounsaturated FA (*p* < 0.05), and FA containing palmitoleic acid (16:1; *p* < 0.01) and linolenic acid (18:3; *p* < 0.001), while diglycerides (DG) and TG were decreased (for TG, *p* < 0.05) (Figure 2). Other lipid classes such as phosphatidylcholines (PC), lysophosphatiylcholines, phosphatidylinositols (*p* < 0.05), lysophosphatidylinositols, phosphatidylethanolamines (PE), lysophosphatidylethanolamines (LPE), or sphingomyelin (SM; *p* < 0.01) were increased in the serum of NAFLD patients with high circulating leptin levels (Figure 2).

Overall, serum leptin predicts NAFLD, correlates with serum lipid changes, and is a potentially valuable tool for NAFLD diagnosis.

### Adiponectin and Specific Lipid Species Distinguish NASH

**Figure 3:**
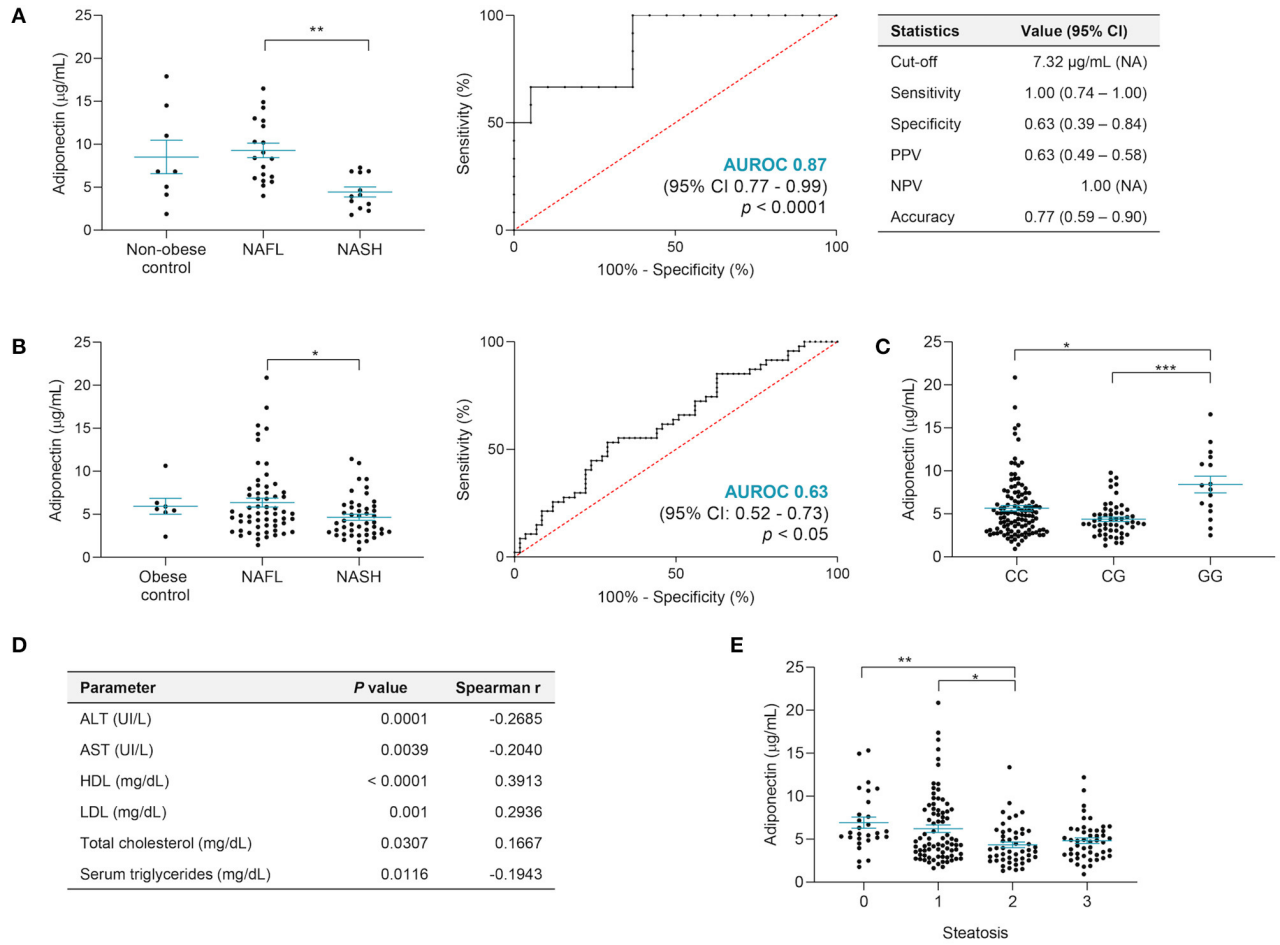
Adiponectin serum levels differentiate NAFL and NASH, but *PNPLA3* genotype may be a confounding factor. **(A)** In the discovery cohort, the adiponectin serum levels were significantly lower in NASH when compared to NAFL, presenting an AUROC value of 0.87 and very high sensitivity and NPV (non-obese control, *n* = 8; NAFL, *n* = 19; NASH, *n* = 12). **(B)** In the validation cohort, adiponectin distinguishes NAFL from NASH only within *PNPLA3* CC allele carriers, with an AUROC value superior to 0.6 obese controls, *n* = 7; NAFL, *n* = 59, NASH, *n* = 47). **(C)** In the validation cohort, NAFLD patients with *PNPLA3* GG genotype also showed increased levels of adiponectin. The *x*-axis represents the three possible genotypes for rs738409 polymorphism: CC allele, *n* = 117; CG allele, *n* = 55; GG allele, *n* = 16). **(D)** Correlations between adiponectin serum levels and several parameters: AST and ALT serum levels (both *n* = 199), HDL and LDL serum levels (both *n* = 167), total cholesterol levels (*n* = 168), and triglyceride serum levels (*n* = 168). **(E)** The adiponectin levels decreased with higher grades of steatosis. The *x*-axis represents the histological grade of steatosis, ranging from 0 to 3. There was a significant difference between grade 2 vs. grade 1 and vs. grade 0 (*n* = 28, grade 0; *n* = 80, grade 1; *n* = 49, grade 2; *n* = 48, grade 3). Adiponectin levels depicted as mean ± SEM. ALT, alanine aminotransferase; AST, aspartate aminotransferase; CI, confidence interval; HDL, high-density lipoprotein; LDL, low-density lipoprotein; NAFL, non-alcoholic fatty liver; NASH, non-alcoholic steatohepatitis. *p < 0.05; **p < 0.01; ***p < 0.001.

In the discovery cohort, adiponectin was similar among men and women (6.55 ± 0.98 vs. 7.79 ± 1.04 μg/ml) and significantly lower in NASH vs. NAFL (non-obese controls: 8.51 ± 1.94 μg/ml; NAFL: 9.23 ± 0.85 μg/ml; NASH: 4.45 ± 0.58 μg/ml; *p* < 0.01) (Figure 3A). Serum adiponectin showed a good assay performance when distinguishing NAFL vs. NASH (AUROC = 0.87; 95% CI, 0.77–0.99; *p* < 0.0001). This allowed us to establish a cutoff value <7.32 μg/ml for diagnosing NASH, with an overall accuracy of 77%, sensitivity of 100%, and NPV (Figure 3A). Additionally, the adiponectin serum levels negatively correlated with the serum markers of liver injury ALT (Spearman *r* = −0.46, *p* < 0.01) and GGT (Spearman *r* = −0.42, *p* < 0.05).

In the validation cohort, adiponectin was lower in men (4.4 ± 0.32 vs. 6.07 ± 0.29 μg/ml, *p* < 0.0001) and, although not significant, were lower in NASH (4.94 ± 0.27 μg/ml) vs. both non-obese (6.21 ± 0.97 μg/ml) and obese (6.56 ± 0.93 μg/ml) controls or NAFL (6.22 ± 0.45 μg/ml). In this cohort, serum adiponectin was only able to distinguish NAFL vs. NASH when NAFLD patients homozygous for *PNPLA3* wild-type allele c.444C were considered, even though with a suboptimal performance (AUROC = 0.63; *p* < 0.05) (Figure 3B). By applying the previously established cutoff value of adiponectin <7.32 μg/ml for NASH diagnosis, the PPV and NPV were 49 and 71%, respectively. This AUROC value was paralleled by AST/ALT ratio and only overcome by MRI or Folch method AUROCs, however with lower specificity values (Table 3). Curiously, NAFLD patients homozygous for *PNPLA3* risk allele c.444G presented significantly higher adiponectin (GG vs. CC, *p* < 0.05; GG vs. CG, *p* < 0.0001; Figure 3C). In the validation cohort, no differences were found between adiponectin and fibrosis stage and ballooning and lobular inflammation, neither for the presence/absence of co-morbidities nor for the other NAFLD risk-associated polymorphisms (Supplementary Figure 2). Adiponectin still negatively correlated with AST (*p* < 0.01) and ALT (*p* < 0.001) and positively correlated with total cholesterol (*p* < 0.05), HDL (*p* < 0.001), and LDL (*p* < 0.01), regardless of *PNPLA3* genotype (Figure 3D). Adiponectin decreased with severity of steatosis and negatively correlated with serum TG (*p* < 0.05; Figures 3D,E).

**Table 3:** Performance of serum adiponectin in the differential diagnosis of NAFL vs. NASH in patients homozygous for the wild-type allele CC in the *PNPLA3* rs738409 polymorphism.

	**Cutoff**	**AUROC (95% CI)**	**Sensitivity (95% CI)**	**Specificity (95% CI)**	**Accuracy (95% CI)**	**PPV (95% CI)**	**NPV (95% CI)**	**TP**	**TN**	**FP**	**FN**
Adiponectin	<7.32 μg/ml	0.63^*^ (0.52–0.73)	0.85 (0.72–0.94)	0.29 (0.18–0.42)	0.54 (0.44–0.64)	0.49 (0.44–0.54)	0.71 (0.52–0.84)	40	17	42	7
AST/ALT	<0.96	0.65^**^ (0.55–0.76)	0.89 (0.77–0.96)	0.49 (0.36–0.63)	0.67 (0.75–0.76)	0.59 (0.52–0.66)	0.85 (0.70–0.93)	42	28	29	5
MRI	>0.15	0.90^****^ (0.69–0.91)	0.85 (0.68–0.95)	0.68 (0.50–0.83)	0.76 (0.64–0.86)	0.72 (0.61–0.81)	0.82 (0.67–0.91)	28	11	5	23
Hepatic TG content evaluated by Folch method	>42.85 mg/g	0.75^***^ (0.63–0.87)	0.83 (0.67–0.94)	0.59 (0.42–0.74)	0.70 (0.59–0.80)	0.64 (0.54–0.72)	0.80 (0.65–0.90)	30	24	17	6

*Applied in validation cohort. Adiponectin; n = 106; AST/ALT, n = 104; MRI, n = 67; hepatic TG content evaluated by Folch method, n = 77. NASH is considered positive if the levels of each parameter are superior or inferior to the cutoff value as indicated*.

*AST/ALT, aspartate aminotransferase to alanine aminotransferase ratio; CI, confidence interval; FN, false negative; FP, false positive; MRI, magnetic resonance imaging; NAFL, nonalcoholic fatty liver; NASH, nonalcoholic steatohepatitis; NPV, negative predictive value; PPV, positive predictive value; TN, true negative; TP, true positive*.

* *p < 0.05*;

** *p < 0.01*;

*** *p < 0.001*;

**** *p < 0.0001*.

Although not significant, higher serum adiponectin positively correlated with serum lipid classes, except for DG, TG, and steroids (Figure 2). Both PE and PC containing arachidonic (20:4) or docosahexaenoic (DHA; 22:6n-3) acids were increased in patients with high circulating adiponectin (*p* < 0.05 and *p* < 0.01). Moreover, the serum levels of 147 metabolites were significantly different between NAFL and NASH, of which 35 were highly correlated with serum adiponectin (Supplementary Table 2). Among those, nine TG were already described as capable of distinguishing NAFL from NASH (16). Sixty-five out of the 147 metabolites presented AUROC >0.60 and were considered for binomial regression analysis. This allowed establishing a panel combining adiponectin and nine serum lipids (mainly TG, PC, and SM), reaching an AUROC value of ~0.80 and an overall accuracy of 74% in distinguishing NAFL from NASH in all patients, regardless of *PNPLA3* genotype. When this panel was applied to homozygous carriers of the *PNPLA3* c.444C allele, the AUROC value increased to 0.83 and the accuracy to 80% (Table 4).

**Table 4:** Performance of serum lipids and combination panel with serum adiponectin in the differential diagnosis of NAFL *vs*. NASH.

	**Cutoff**	**AUROC (95% CI)**	**Sensitivity (95% CI)**	**Specificity (95% CI)**	**Accuracy (95% CI)**	**PPV (95% CI)**	**NPV (95% CI)**	**TP**	**TN**	**FP**	**FN**
TG(44:0)	>0.25	0.72^*^ (0.65–0.79)	0.60 (0.49–0.70)	0.75 (0.65–0.83)	0.67 (0.60–0.74)	0.70 (0.61–0.77)	0.65 (0.59–0.71)	54	68	23	36
TG(49:2)	>0.71	0.70^*^ (0.63–0.78)	0.71 (0.61–0.80)	0.58 (0.47–0.69)	0.65 (0.57–0.72)	0.63 (0.56–0.69)	0.67 (0.59–0.75)	64	53	38	26
TG(51:3)	>1.02	0.61^*^ (0.54–0.69)	0.61 (0.50–0.71)	0.56 (0.45–0.66)	0.59 (0.51–0.66)	0.58 (0.51–0.65)	0.59 (0.52–0.67)	55	51	40	35
TG(52:1)	>0.85	0.75^**^ (0.68–0.82)	0.79 (0.69–0.87)	0.59 (0.49–0.70)	0.69 (0.62–0.76)	0.66 (0.59–0.72)	0.74 (0.65–0.81)	71	54	37	19
TG(53:1)	>0.48	0.74^*^ (0.67–0.81)	0.89 (0.81–0.95)	0.49 (0.39–0.60)	0.69 (0.62–0.76)	0.63 (0.58–0.68)	0.82 (0.71–0.89)	80	45	46	10
TG(54:0)	>0.38	0.68^*^ (0.61–0.76)	0.72 (0.62–0.81)	0.57 (0.46–0.67)	0.65 (0.57–0.72)	0.63 (0.56–0.69)	0.68 (0.59–0.75)	65	52	39	25
TG(60:3)	>0.58	0.60 (0.50–0.66)	0.70 (0.59–0.79)	0.47 (0.37–0.58)	0.59 (0.51–0.66)	0.57 (0.51–0.62)	0.61 (0.52–0.70)	63	43	48	27
PC(0:0/14:0)	>0.22	0.70^*^ (0.62–0.77)	0.80 (0.70–0.88)	0.51 (0.40–0.61)	0.65 (0.58–0.72)	0.62 (0.56–0.67)	0.72 (0.62–0.80)	72	46	45	18
SM(38:0)	>1.16	0.71^*^ (0.63–0.78)	0.74 (0.64–0.83)	0.64 (0.53–0.74)	0.69 (0.62–0.76)	0.67 (0.60–0.73)	0.72 (0.63–0.79)	67	58	33	23
Adiponectin + 9 lipids	-	0.796^**^ (0.73–0.86)	0.71 (0.61–0.80)	0.77 (0.67–0.85)	0.74 (0.67–0.80)	0.75 (0.67–0.82)	0.73 (0.66–0.79)	64	70	21	26
Adiponectin + 9 lipids (*PNPLA3* rs738409 CC)	-	0.832^**^ (0.75–0.91)	0.70 (0.55–0.83)	0.88 (0.77–0.95)	0.802 (0.71–0.87)	0.83 (0.70–0.91)	0.79 (0.70–0.85)	33	52	7	14

*Applied in the validation cohort, n = 181; PNPLA3 rs738409 CC carriers, n = 106. NASH is considered as positive if the levels of each parameter are superior or inferior to the cutoff value as indicated; for adiponectin, <7.32 μg/ml. p-values corrected with Benjamini–Hochberg procedure, assuming 5% false discovery rate*.

*CI, confidence interval; FN, false negative; FP, false positive; NAFL, nonalcoholic fatty liver; NASH, nonalcoholic steatohepatitis; NPV, negative predictive value; PC, phosphatidylcholine; PPV, positive predictive value; TG, triglyceride; TN, true negative; TP, true positive; SM, sphingomyelin*.

* *p < 0.05*;

** *p < 0.01*.

Overall, serum adiponectin inversely correlated with liver injury markers and, when combined with nine specific lipids, distinguished NAFL from NASH patients with high accuracy.

### IGF-1 Predicts Advanced Fibrosis in NAFLD

**Figure 4:**
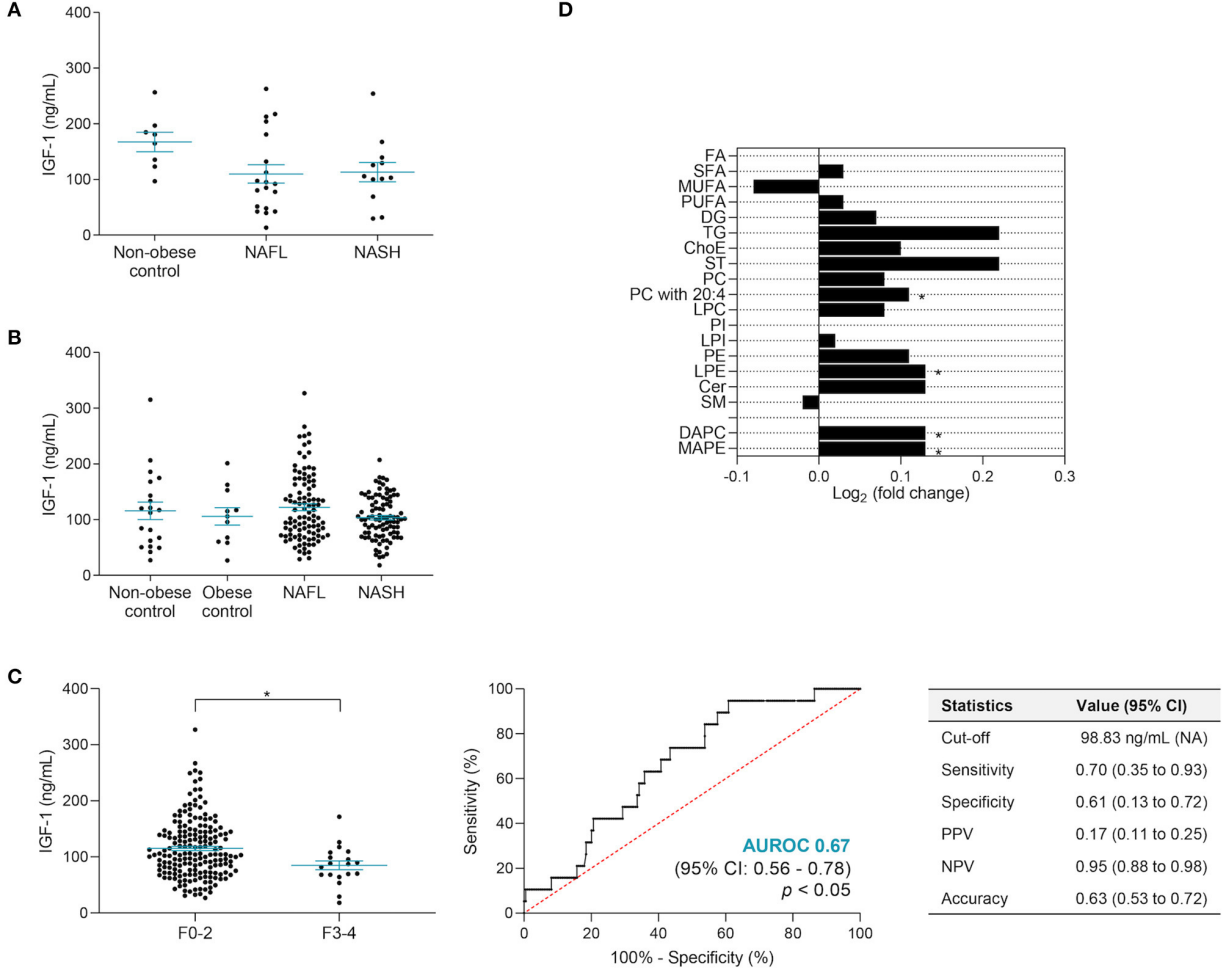
IGF-1 serum levels correlated with liver fibrosis score. **(A)** In the discovery cohort, the IGF-1 serum levels were slightly lower in NASH patients compared to NAFL and non-obese contros. **(B)** No significant differences in IGF-1 levels were found among controls and patients with NAFLD in the validation cohort. **(C)** In the validation cohort, NAFLD patients with advanced fibrosis presented significantly lower levels of IGF-1. The *x*-axis represents fibrosis score dichotomy: none to moderate fibrosis (F0-2; *n* = 184) and advanced fibrosis to cirrhosis (F3-4; *n* = 19). IGF-1 alone presents an AUROC value of 0.67 when distinguishing F0-2 *vs*. F3-4. **(D)** Serum lipidomic signature associated with higher IGF-1 circulating levels. Data presented as log_2_ of the fold change between higher *vs*. lower than the median IGF-1 level (103.65 ng/ml). Higher, *n* = 97; lower, *n* = 97. IGF-1 levels depicted as mean ± SEM. Cer, ceramide; ChoE, cholesteryl ester; CI, confidence interval; DAPC, diacylglycerophosphocholine; DG, diglyceride; FA, fatty acid; IGF-1, insulin-like growth factor 1; LPC, lysophosphatidylcholines; LPE, lysophosphatidylethanolamines; LPI, lysophosphatidylinositols; MAPE, monoacylglycerophosphoethanolamine; MUFA, monounsaturated FA; NAFL, non-alcoholic fatty liver; NASH, non-alcoholic steatohepatitis; NPV, negative predictive value; PPV, positive predictive value; PC, phosphatidylcholines; PE, phosphatidylethanolamines; PI, phosphatidylinositols; PUFA, polyunsaturated FA; SFA, saturated FA; SM, sphingomyelins; ST, steroids; TG, triglycerides; 20:4, arachidonic acid. *p < 0.05.

IGF-1 was higher in men in both cohorts (discovery cohort: 152.08 ± 20.78 vs. 81.04 ± 12.24 ng/ml, *p* < 0.01; validation cohort: 126.5 ± 6.78 vs. 103.4 ± 3.87 ng/ml, *p* < 0.01). In the discovery cohort, the IGF-1 levels decreased in NAFL (109.95 ± 16.55 ng/ml) and NASH (113.19 ± 17.39 ng/ml) when compared to non-obese controls (167.42 ± 17.55 ng/ml), although not significantly. In the validation cohort, there were no significant changes in IGF-1 between non-obese controls (115.75 ± 15.69 ng/ml), obese controls (105.83 ± 15.62 ng/ml), NAFL (122.30 ± 6.05 ng/ml), and NASH (103.50 ± 3.98 ng/ml), although an inverse correlation with BMI was observed (*n* = 199; Spearman *r* = −0.1647; 95% CI, 0.30-−0.02; *p* < 0.05) (Figures 4A,B). In agreement, in the validation cohort, there were also no significant differences in IGF-1 relating to liver steatosis, lobular inflammation, or hepatocyte ballooning severity (Supplementary Figure 3), yet IGF-1 was significantly lower in NAFLD patients with advanced fibrosis (F3–4; *p* < 0.05) (Figure 4C). By establishing a cutoff value of IGF-1 inferior to 98.83 ng/ml for the presence of advanced fibrosis, 70% sensitivity, 61% specificity, and 63% overall accuracy were obtained (Figure 4C and Table 5). Overall, IGF-1, as well as the pre-established biomarker fibrosis panels APRI, Fib-4, and NFS, displayed a suboptimal performance in F0–2 vs. F3–4 discrimination. However, by combining serum IGF-1, ferritin, and INR, the discriminatory power was significantly improved, resulting in AUROC value of 0.81 and overall accuracy of 93%, considering the ~10% prevalence of advanced fibrosis in this cohort (Table 5). Importantly, besides a robust NPV, this panel displayed 71% PPV value, contrasting with the low PPV values from APRI, Fib-4, and NFS. Circulating IGF-1 did not correlate with other parameters or co-morbidities (Supplementary Figure 3).

**Table 5:** Performance of IGF-1, alone or in a combination panel, and classical fibrosis scores in distinguishing F0-2 vs. F3-4 grades of fibrosis.

	**Cutoff**	**AUROC (95% CI)**	**Sensitivity (95% CI)**	**Specificity (95% CI)**	**Accuracy (95% CI)**	**PPV (95% CI)**	**NPV (95% CI)**	**TP**	**TN**	**FP**	**FN**
IGF-1	<98.83 ng/ml	0.67^*^ (0.56–0.78)	0.70 (0.35–0.93)	0.61 (0.51–0.72)	0.63 (0.53–0.72)	0.17 (0.11–0.25)	0.95 (0.88–0.98)	7	60	37	3
APRI	>0.31	0.62 (0.43–0.81)	0.60 (0.26–0.88)	0.71 (0.61–0.80)	0.70 (0.60–0.79)	0.18 (0.11–0.28)	0.95 (0.89–0.97)	6	69	28	4
Fib-4	>0.75	0.66 (0.49–0.82)	0.90 (0.56–0.99)	0.47 (0.37–0.58)	0.51 (0.42–0.61)	0.15 (0.12–0.19)	0.98 (0.88–0.99)	9	46	51	1
NFS	>-0.96	0.74 (0.52–0.89)	0.43 (0.10–0.82)	0.88 (0.77–0.94)	0.83 (0.72–0.91)	0.28 (0.12–0.53)	0.93 (0.88–0.96)	3	56	8	4
Ferritin	>118 ng/ml	0.68^*^ (0.49–0.87)	0.8 (0.44–0.97)	0.65 (0.55–0.74)	0.66 (0.57–0.75)	0.19 (0.13–0.26)	0.97 (0.90–0.99)	8	63	34	2
INR	>1.03	0.78^*^ (0.64–0.93)	0.70 (0.35–0.93)	0.85 (0.76–0.91)	0.83 (0.75–0.90)	0.32 (0.20–0.46)	0.96 (0.91–0.99)	7	82	15	3
IGF-1 + ferritin + INR	-	0.81^*^ (n.d.)	0.50 (0.19–0.81)	0.98 (0.93–0.99)	0.93 (0.87–0.97)	0.71 (0.37–0.91)	0.95 (0.91–0.97)	5	95	2	5

*Applied in the validation cohort. NSF, n = 71; others, n = 107. F3-4 is considered as positive if the levels of each parameter are superior or inferior to the cutoff value as indicated. p-values corrected with Benjamini–Hochberg procedure, assuming 5% false discovery rate*.

*APRI, aspartate aminotransferase-to-platelet ratio; CI, confidence interval; FN, false negative; FP, false positive; IGF-1, insulin-like growth factor 1; INR, international normalized ratio; NFS, NAFLD fibrosis score; NPV, negative predictive value; PPV, positive predictive value; TN, true negative; TP, true positive; n.d., not determined due to the low number of F3-4 patients*.

* *p < 0.05*.

Serum metabolomic analysis showed that higher IGF-1 was associated with an overall increase in main lipid classes, significant for LPE (*p* < 0.05), PC containing arachidonic acid (20:4; *p* < 0.05), diacylglycerophosphocholine (*p* < 0.05), and monoacylglycerophosphoethanolamine (*p* < 0.05) (Figure 4D). Moreover, 26 specific serum metabolites were able to distinguish advanced fibrosis, with several bile acids increased in advanced fibrosis, and both cholic acid and PC (17:0/18:2) positively correlated with IGF-1 (Supplementary Table 3).

IGF-1 altogether could identify advanced fibrosis, particularly in combination with INR and ferritin, and correlates with serum lipid changes associated with fibrosis.

## Discussion

The strong association between obesity and NAFLD, parallel with the continuous rise in the prevalence of obesity, prompts a more detailed study of the role of adipokines and other hormones as potential non-invasive biomarkers for NAFLD (1). Therefore, we measured serum leptin, adiponectin, and IGF-1 in two independent cohorts, comprising both biopsy-proven healthy-liver controls as well as obese NAFL and NASH patients. In the discovery cohort, the serum hormones were correlated with clinical and histological information, including NAS, fibrosis stage, and biochemical parameters. Results were then validated in a second cohort, which further allowed us to explore the role of obesity as a confounding factor by including a control group of obese healthy-liver individuals. Additionally, in this validation cohort, hormone serum levels were correlated with liver imaging, co-morbidities, and NAFLD risk-associated polymorphisms. For a subset of patients, serum metabolomic analysis was also performed. This strategy showed that particularly leptin and adiponectin can be potential diagnosis and stratification biomarkers in NAFLD. The role of these two adipokines in NAFLD pathophysiology, diagnosis, and even treatment has attracted significant attention despite the controversial results.

Leptin is a 16-kDa pro-inflammatory adipokine produced by adipocytes. Serum leptin levels reflect total body mass. Leptin may exert an anti-steatotic action in early NAFLD by promoting FA oxidation and decreased lipogenesis and a pro-inflammatory and pro-fibrotic action at later disease stages by increasing hepatic reactive oxygen species generation, pro-inflammatory cytokine release, and enhanced fibrinogenesis (28, 29). Previous reports have generally found increased serum leptin in NAFLD patients (12, 29, 30). Still the role of obesity as a bias remains poorly explored. Our results in the discovery cohort showed that leptin accurately identified NAFLD patients, where there was no correlation with BMI. Moreover, in the validation cohort, although leptin levels positively correlated with BMI, they were higher in NAFL and NASH compared with both non-obese and obese controls, suggesting that the association between NAFLD and leptin is more robust than the effect of obesity on leptin levels. In this cohort, serum leptin showed again a very good performance as a potential biomarker to distinguish NAFLD patients from healthy individuals. After applying the cutoff value of leptin at >9.33 ng/ml for diagnosing NAFLD in the validation cohort, despite having a lower general accuracy, it still presented sensitivity, specificity, and NPV values far superior than those previously reported (31, 32).

In turn, adiponectin, a 30-kDa protein mainly secreted by adipose tissue, is the most well-studied adipokine in the pathogenesis of NAFLD (30, 33). It is considered to have anti-inflammatory, anti-steatotic, and anti-fibrotic effects. In the liver, adiponectin prevents lipid accumulation by promoting FA oxidation *via* peroxisome proliferator-activated receptor alpha (33). Adiponectin also downregulates pro-inflammatory cytokines produced by Kupffer cells and hepatic stellate cells, thus inhibiting their transformation into myofibroblasts and consequently decreasing liver fibrosis (30, 34–36). Several reports have shown a reverse relation between adiponectin circulating levels and body fat mass and its decrease in obesity, type 2 diabetes, insulin resistance, and dyslipidemia (30, 34). A meta-analysis showed that adiponectin decreases in healthy controls compared to NAFL, and in the latter compared to NASH (37). However, there is significant heterogeneity among studies, and the importance of including biopsy-proven healthy controls is underlined; most differences in adiponectin between individuals with NAFLD and controls were observed when the controls were not subjected to liver biopsy (37). In the discovery cohort, adiponectin presented a very promising performance in distinguishing NAFL vs. NASH. However, when tested in the larger validation cohort, adiponectin only presented a fair discriminatory power in this stratification when homozygous carriers of the *PNPLA3* c.444C allele were considered. Importantly, the discriminatory power of adiponectin regarding NAFL vs. NASH patients was improved when combined with nine specific lipids. Previously, others have shown that combining adiponectin with homeostatic model assessment for insulin resistance and type IV collagen 7S increased the assay sensitivity from 68 to 94% in early-stage NASH prediction (38), while combining adiponectin, resistin, and cleaved cytokeratin-18 predicted NASH with an AUROC of 0.90 (39). Similar approaches combining adiponectin serum levels with a panel of serum lipids, mainly PC species, have been shown to discriminate between healthy individuals, NAFL, and NASH with high accuracy (40). Curiously, previous observations showed that NAFLD patients homozygous for the c.444G allele presented decreased adiponectin (41); however, the opposite was observed here. Nonetheless, these results could explain how this subgroup of NAFLD patients has an improved response to treatment and lifestyle interventions, including bariatric surgery, when compared to CG and CC carriers (42). Additionally, we showed that lower levels of serum adiponectin associated with higher AST and ALT, thus suggesting a role of adiponectin in preventing liver damage. Overall, our observations suggest that adiponectin might be a valuable tool in NAFL vs. NASH stratification; however, its role as a biomarker in NAFLD can be challenged by risk-conferring genetic variants, such as the *PNPLA3* c.444G allele, and can benefit from combination panels, such as the one presented comprising serum lipids.

In fact, NAFLD has been associated with increased lipids in both liver tissue and plasma. Here we observed that higher circulating leptin associated with increased serum lipid profile and increased hepatic steatosis as assessed by histology and imaging. Similarly, higher circulating adiponectin associated with an increased serum lipid profile. The fact that adiponectin is inversely correlated with liver fat contents, as evaluated by MRI, might suggest a protective action for adiponectin by promoting lipid removal from the liver into circulation. For instance, higher adiponectin associated with higher serum PE containing arachidonic acid (C20:4n-6), while lower liver levels of this acid have been observed in NAFLD due to active conversion into pro-inflammatory prostaglandins (13). Furthermore, increased serum PE containing DHA also followed higher adiponectin. DHA, whose lower levels have been associated with both NAFL and NASH, is believed to have anti-inflammatory and metabolic effects and to be able to lower liver TG (13, 14, 43, 44). This might also explain why higher adiponectin inversely correlates with TG as measured by blood biochemistry and serum metabolomics.

In turn, IGF-1, mainly produced in the liver in response to growth hormone stimulation, is known to regulate insulin sensitivity and decrease hepatic TG accumulation in the liver (45). While a few clinical studies have associated low serum IGF-1 with increased severity of steatosis (46, 47) or lobular inflammation (48), our results and those of others (49) failed to show any association. On the other hand, clinical studies are consensual when reporting low serum IGF-1 association with the severity of fibrosis (46, 48–50). In fact, *in vitro* and *in vivo* studies showed that IGF-1 can induce cellular senescence and hepatic stellate cell inactivation through a p53-dependent manner, thus improving experimental fibrosis (51). Accordingly, here we showed that patients with NAFLD and advanced fibrosis presented significantly lower serum IGF-1. More importantly, we found that the 63% accuracy of serum IGF-1 alone in distinguishing low/mild fibrosis from advanced fibrosis is further improved to 93% if IGF-1 is combined with ferritin and INR. These results are very similar to those determined for the Enhanced Liver Fibrosis (ELF) test, recommended by the NICE guidelines for the evaluation of fibrosis in NAFLD (9). A poor PPV value has still been reported for the generality of fibrosis tests, including ELF (52). The panel here proposed displayed high PPV value, being matched by the recent Hepamet fibrosis scoring system where a PPV of 76% was achieved (53). Recent reports suggest that IGF-1 biodisponibility might be decreased during disease progression toward NASH and liver fibrosis and advance IGF-1/intact IGFBP3 ratio as a fibrosis predictor (54). Moreover, IGF-1 might associate with a fibrosis-specific serum metabolite profile, where many bile acids were found increased in advanced fibrosis, namely, cholic acid, which also correlated with serum IGF-1. Increased levels of many bile acids, including cholic acid, have already been associated with liver fibrosis (55).

Inclusion of a patient group comprising lean-NAFLD would allow further inquiries on adipokines as NAFLD biomarkers, particularly for leptin. It is also recognized that the number of healthy non-obese controls is relatively small, mostly due to the difficulty in obtaining biopsies from healthy-liver individuals. Lastly, the performance of the proposed biomarker panels comprising serum adiponectin and lipids for NAFL vs. NASH stratification and serum IGF-1 with INR and ferritin for identification of advanced fibrosis should be prospectively tested in a different cohort.

## Conclusion

In conclusion, we suggest that serum leptin can identify NAFLD without obesity as a confounding factor, whereas adiponectin combined with specific serum lipids might be advantageous for NAFL vs. NASH stratification. In turn, IGF-1, together with ferritin and INR, could embody a valuable biomarker panel to identify advanced fibrosis, which could be easily implemented in clinics and presents a better PPV than the regularly used algorithms. Serum metabolomics correlate with hormone levels and offer new opportunities for improved non-invasive biomarker panels.

## Data Availability Statement

The data that support the current study are available within the article/Supplementary Materials, and from the corresponding author upon reasonable request.

## Ethics Statement

The studies involving human participants were reviewed and approved by sample collection was performed after patient informed consent and Institutional Review Board approval, in accordance with the Declaration of Helsinki, by the ethics committees of Hospital de Santa Maria (Lisbon, Portugal) and Donostia University Hospital (San Sebastian, Spain). The patients/participants provided their written informed consent to participate in this study.

## Author Contributions

VM contributed to the methodology, validation, formal analysis, investigation, writing—original draft, writing—review and editing, and visualization. MA contributed to the supervision, methodology, writing–review and editing, and visualization. NB, MP, RL, AC, EA, and JA conducted the investigation. FD-R contributed to the formal analysis, writing—review, and editing. AS-L contributed to data curation, writing—review, and editing. RJ-A and EE contributed to data curation. LB, DT, and BF contributed to writing—review and editing. MM contributed to data curation. CA, MK, and FL contributed to the investigation and writing—review and editing. HC-P contributed to the resources, data curation, and writing—review and editing. JB contributed to the resources, data curation, supervision, funding acquisition, writing—review, and editing. RC contributed to writing—review and editing. AN contributed to the conceptualization, resources, writing—review, and editing. CR contributed to the supervision, conceptualization, funding acquisition, resources, writing—review, and editing. All the authors read and approved the final manuscript.

## Conflict of Interest

NB, BF, and AN are employees at Mediagnost, GmbH. CA and EA are employees at One Way Liver, S.L. (OWL Metabolomics). JB is scientific advisor for OWL Metabolomics. The remaining authors declare that the research was conducted in the absence of any commercial or financial relationships that could be construed as a potential conflict of interest.

## Abbreviations

ALT: alanine aminotransferase
AHT: arterial hypertension
APRI: AST/platelet ratio index
AST: aspartate aminotransferase
AUROC: area under the ROC curve
DG: diglycerides
DHA: docosahexaenoic
ELF: enhanced liver fibrosis
ELISA: enzyme-linked immunosorbent assay
FA: fatty acids
Fib-4: fibrosis-4 index
GGT: gamma-glutamyl transferase
HDL: high-density lipoprotein
IGF-1: insulin-like growth factor 1
INR: international normalized ratio
LDL: low-density lipoprotein
LPC: lysophosphatiylcholines
LPE: lysophosphatidylethanolamines
LPI: lysophosphatidylinositols
MRI: magnetic resonance imaging
NAFL: non-alcoholic fatty liver
NAFLD: non-alcoholic fatty liver disease
NAS: NAFLD activity score
NASH: non-alcoholic steatohepatitis
NFS: NAFLD fibrosis score
NPV: negative predictive value
PC: phosphatidylcholines
PE: phosphatidylethanolamines
PI: phosphatidylinositols
PPV: positive predictive value
ROC: uniparameter receiver operating characteristic
SM: sphingomyelin
TG: triglycerides.
