# Supplementary material for: Adiponectin, Leptin, and IGF-1 Are Useful Diagnostic and Stratification Biomarkers of NAFLD
Source: Front Med (Lausanne). 2021 Jun 23;8:683250. doi: 10.3389/fmed.2021.683250 (PMC8260936; doi:10.3389/fmed.2021.683250)
Supplement: Supplementary file 1 [file Data_Sheet_1.pdf]

Supplementary Material

Adiponectin, leptin and IGF-1 are useful diagnostic and stratification biomarkers of NAFLD

Vanda Marques[1], Marta B. Afonso[1], Nina Bierig[2], Filipa Duarte-Ramos[1,3], Álvaro Santos-Laso[4], Raul Jimenez-Agüero[4], Emma Eizaguirre[4], Luis Bujanda[4,5], Maria J. Pareja[6], Rita Luís[7], Adília Costa[7], Mariana V. Machado[8,9], Cristina Alonso[10], Enara Arretxe[10], José M. Alustiza[4,11], Marcin Krawczyk[12,13], Frank Lammert[12], Dina G. Tiniakos[14,15], Bertram Flehmig[2], Helena Cortez-Pinto[8,9], Jesus M. Banales[4,5,16], Rui E. Castro[1], Andrea Normann[2], Cecília M. P. Rodrigues[1*]

[1]Research Institute for Medicines (iMed.ULisboa), Faculty of Pharmacy, Universidade de Lisboa, Lisbon, Portugal

[2]Mediagnost, GmbH, Reutlingen, Germany

[3]EPIUnit - Instituto de Saúde Pública, Universidade do Porto, Oporto, Portugal

[4]Department of Liver and Gastrointestinal Diseases, Biodonostia Health Research Institute, Donostia University Hospital, University of the Basque Country (UPV/EHU), San Sebastian, Spain

[5]National Institute for the Study of Liver and Gastrointestinal Diseases (CIBERehd, Instituto de Salud Carlos III), Madrid, Spain

[6]Hospital de Valme, Sevilla, Spain

[7]Pathological Anatomy, Hospital de Santa Maria, Centro Hospitalar Universitário Lisboa Norte, Lisbon, Portugal

[8]Clinica Universitária de Gastrenterologia, Faculdade de Medicina, Universidade de Lisboa, Lisbon, Portugal

[9]Departamento de Gastrenterologia, Centro Hospitalar Universitário Lisboa Norte, Lisbon, Portugal

[10]OWL Metabolomics, Bizkaia Technology Park, Derio, Spain

[11]Radiology Service, Osatek, Donostia, Spain

[12]Department of Medicine II, Saarland University Medical Center, Homburg, Germany

[13]Laboratory of Metabolic Liver Diseases, Department of General, Transplant and Liver Surgery, Centre for Preclinical Research, Medical University of Warsaw, Warsaw, Poland

[14]Translational & Clinical Research Institute, Faculty of Medical Sciences, Newcastle University, Newcastle upon Tyne, UK

[15]Department of Pathology, Aretaieio Hospital, National and Kapodistrian University of Athens, Athens, Greece

[16]IKERBASQUE, Basque Foundation for Science, Bilbao, Spain

1 Supplementary Tables

Table S1. Histology data of patients in validation cohort.

	NAFL (n = 100)	NASH (n = 94)
**Steatosis**		
0 (< 5%)	16 (16.0%)	0 (0.0%)
1 (5 – 33%)	68 (68.0%)	12 (12.8%)
2 (34 – 66%)	16 (16.0%)	34 (36.2%)
3 (> 66%)	0 (0.0%)	48 (51.1%)
**Lobular inflammation**		
0 (none)	6 (6.0%)	0 (0.0%)
1 (< 2 foci)	64 (64.0%)	19 (20.2%)
2 (2 – 4 foci)	28 (28.0%)	45 (47.9%)
3 (> 4 foci)	2 (2.0%)	30 (31.9%)
**Ballooned hepatocytes**		
0 (none)	21 (21.0%)	2 (2.1%)
1 (few)	58 (58.0%)	34 (36.2%)
2 (many)	21 (21.0%)	58 (61.7%)
**Fibrosis stage**		
F0-2 (none to moderate)	90 (90.0%)	81 (86.2%)
F3-4 (severe to cirrhosis)	8 (8.0%)	10 (10.6%)

Steatosis, lobular inflammation and ballooned hepatocytes were assessed according to NASH Clinical Research Network(1). Steatosis is expressed as % of affected hepatocytes. Lobular inflammation is expressed as necroinflammation foci per x20 optical field.

Table S2. Serum metabolites able to distinguish NAFL from NASH.

	Serum lipidomics			Correlation with serum adiponectin
	NAFL	NASH	AUROC	
Arachidyl carnitine	0.34 ± 0.02†	0.28 ± 0.01	0.457	0.097
AC(14:2n-x)	1.13 ± 0.06†	0.97 ± 0.06	0.410	0.012
Deoxycholic acid	0.27 ± 0.05‡	0.34 ± 0.03	0.656**	-0.094
Taurocholic acid	1.63 ± 1.26†	0.57 ± 0.18	0.568	0.235**
Glycocholic acid	0.45 ± 0.25‡	0.39 ± 0.14	0.580	0.169*
Cer(d18:1/20:0)	0.88 ± 0.03§	1.09 ± 0.04	0.596	-0.039
Cer(d18:1/22:0)	0.71 ± 0.03§	0.89 ± 0.04	0.647*	-0.034
Cer(d18:1/24:0)	0.79 ± 0.04‡	0.94 ± 0.04	0.618*	0.028
ChoE(16:1)	0.86 ± 0.05§	1.13 ± 0.06	0.590	0.164*
ChoE(20:5)	1.57 ± 0.19†	1.90 ± 0.25	0.518	0.041
PC(32:1)	1.15 ± 0.07§	1.59 ± 0.08	0.658**	0.021
PC(14:0/18:2)	0.60 ± 0.04†	0.74 ± 0.04	0.572	0.054
PC(16:0/18:1)	1.16 ± 0.03‡	1.26 ± 0.03	0.595	0.068
PC(14:0/20:4)	0.76 ± 0.06‡	0.94 ± 0.06	0.577	0.058
PC(18:0/18:1)	0.88 ± 0.04§	1.05 ± 0.04	0.626*	0.041
PC(18:0/18:2)	0.82 ± 0.02†	0.89 ± 0.02	0.554	0.120
PC(16:0/20:5)	1.46 ± 0.17†	1.85 ± 0.27	0.526	0.029
PC(18:3/18:3)	1.38 ± 0.11‡	1.70 ± 0.12	0.535	0.073
PC(18:0/20:3)	1.07 ± 0.05§	1.32 ± 0.06	0.583	0.000
PC(38:5)	1.37 ± 0.10†	1.65 ± 0.15	0.557	0.036
PC(18:0/22:4)	0.90 ± 0.04†	1.10 ± 0.06	0.604	-0.004
PC(40:5)	1.10 ± 0.05‡	1.28 ± 0.06	0.606	0.039
PC(34:3)	0.47 ± 0.03§	0.61 ± 0.04	0.602	0.024
PC(36:3)	0.90 ± 0.03†	1.01 ± 0.03	0.542	0.118
PC(30:0)	0.53 ± 0.05§	0.77 ± 0.05	0.625*	0.003
PE(16:0/18:1)	1.78 ± 0.11†	1.99 ± 0.09	0.596	0.043
PE(16:0/18:2)	0.93 ± 0.04‡	1.11 ± 0.05	0.577	0.055
PC(16:0/17:0)	0.78 ± 0.04†	0.87 ± 0.03	0.540	0.164*
PC(33:1)	0.96 ± 0.04§	1.14 ± 0.04	0.596	0.105
PE(16:0/20:4)	1.55 ± 0.07†	1.79 ± 0.08	0.567	0.078
PC(15:0/20:3)	0.81 ± 0.05†	1.10 ± 0.08	0.555	0.009
PE(18:0/20:4)	1.55 ± 0.06†	1.81 ± 0.08	0.580	0.045
PE(16:0/22:6)	3.16 ± 0.18†	4.07 ± 0.27	0.524	0.139
DG(32:0)	0.40 ± 0.04§	0.58 ± 0.04	0.734***	-0.160*
DG(32:1)	0.34 ± 0.04§	0.52 ± 0.04	0.698***	-0.142*
DG(34:0)	0.74 ± 0.04§	0.97 ± 0.05	0.682***	-0.196**
DG(34:1)	0.43 ± 0.03§	0.59 ± 0.03	0.694***	-0.166*
DG(34:2)	0.41 ± 0.03§	0.54 ± 0.03	0.661**	-0.194**
Stearic acid (18:0)	1.09 ± 0.02§	1.23 ± 0.03	0.635*	-0.025
18:1n-x	0.14 ± 0.01‡	0.19 ± 0.02	0.607	-0.013
Arachidic acid (20:0)	0.72 ± 0.02†	0.78 ± 0.02	0.578	0.039
9,10-DiHOME	1.44 ± 0.16†	0.92 ± 0.10	0.431	0.059
12,13-DiHOME	0.89 ± 0.09†	0.58 ± 0.06	0.429	0.098
PC(14:0/0:0)	0.29 ± 0.02§	0.37 ± 0.02	0.602	0.007
PC(0:0/14:0)	0.26 ± 0.02§	0.35 ± 0.02	0.643*	-0.003
PC(16:0/0:0)	0.72 ± 0.01†	0.75 ± 0.01	0.508	-0.062
PC(0:0/16:0)	0.63 ± 0.01†	0.66 ± 0.01	0.540	-0.020
PC(0:0/16:1)	0.74 ± 0.04§	0.93 ± 0.04	0.608	0.130
PC(0:0/18:0)	0.54 ± 0.01§	0.61 ± 0.01	0.572	-0.054
PC(18:0/0:0)	0.68 ± 0.01§	0.74 ± 0.01	0.576	-0.081
PC(18:1/0:0)	0.09 ± 0.01†	0.10 ± 0.01	0.552	0.093
PC(18:2/0:0)	0.43 ± 0.03§	0.56 ± 0.03	0.628*	0.036
PC(0:0/18:3)	0.37 ± 0.03†	0.45 ± 0.04	0.560	0.107
PC(18:3/0:0)	0.40 ± 0.03†	0.46 ± 0.03	0.561	0.134
PC(18:3/0:0)	0.95 ± 0.08§	1.25 ± 0.07	0.625*	-0.011
PC(20:2/0:0)	1.65 ± 0.13§	2.13 ± 0.12	0.583	0.110
PC(0:0/20:3)	3.64 ± 0.16‡	4.40 ± 0.21	0.534	-0.033

PC(20:3/0:0)	1.60 ± 0.05†	1.73 ± 0.06	0.525	0.043
PC(20:3/0:0)	1.71 ± 0.09†	1.90 ± 0.10	0.518	0.110
PC(0:0/20:5)	1.70 ± 0.17†	2.29 ± 0.35	0.543	0.028
PC(20:5/0:0)	1.45 ± 0.09†	1.78 ± 0.15	0.533	0.054
PC(0:0/22:4)	1.52 ± 0.07†	1.73 ± 0.07	0.623*	-0.041
PC(22:4/0:0)	2.27 ± 0.11†	2.62 ± 0.12	0.611	-0.016
PC(0:0/22:5)	1.45 ± 0.09§	1.83 ± 0.15	0.591	-0.010
PC(17:1/0:0)	0.54 ± 0.02§	0.66 ± 0.03	0.570	0.079
PE(0:0/16:0)	0.51 ± 0.02†	0.57 ± 0.02	0.521	0.070
PE(0:0/16:1)	0.65 ± 0.04§	0.89 ± 0.04	0.691**	-0.013
PE(0:0/18:0)	0.36 ± 0.01§	0.45 ± 0.02	0.632*	0.006
PE(18:0/0:0)	0.45 ± 0.02§	0.52 ± 0.02	0.577	-0.022
PE(18:3/0:0)	0.37 ± 0.03‡	0.43 ± 0.02	0.581	-0.039
PE(0:0/20:3)	0.72 ± 0.04§	0.93 ± 0.04	0.602	-0.068
PE(20:3/0:0)	0.60 ± 0.03§	0.75 ± 0.03	0.580	-0.059
PE(20:4/0:0)	0.81 ± 0.02†	0.88 ± 0.02	0.593	-0.053
LPE(20:5)	0.59 ± 0.08‡	0.80 ± 0.13	0.578	-0.005
PE(20:5/0:0)	0.55 ± 0.07‡	0.71 ± 0.11	0.550	0.000
PE(22:4/0:0)	0.79 ± 0.04†	0.91 ± 0.05	0.598	-0.131
PE(22:5/0:0)	0.46 ± 0.02‡	0.54 ± 0.02	0.575	-0.040
PE(22:5/0:0)	0.57 ± 0.03‡	0.66 ± 0.03	0.565	0.002
PE(22:5/0:0)	0.83 ± 0.05†	1.01 ± 0.06	0.584	-0.020
PE(0:0/22:6)	1.61 ± 0.06‡	1.89 ± 0.08	0.526	0.074
PE(22:6/0:0)	1.49 ± 0.05†	1.66 ± 0.06	0.498	0.088
LPI(16:0)	0.96 ± 0.05§	1.18 ± 0.06	0.583	0.091
LPI(18:0)	0.91 ± 0.03†	1.03 ± 0.04	0.545	0.066
LPI(18:1)	0.92 ± 0.06‡	1.07 ± 0.06	0.567	0.156*
LPI(18:1)	0.99 ± 0.07‡	1.26 ± 0.09	0.571	0.103
PC(O-18:1/22:4)	1.77 ± 0.05‡	1.57 ± 0.04	0.350	0.223**
PC(O-22:1/20:4)	1.47 ± 0.06†	1.31 ± 0.06	0.352	0.166*
PC(O-24:1/20:4)	1.74 ± 0.07†	1.55 ± 0.07	0.371	0.178*
PE(18:1e/22:6)	1.76 ± 0.12†	2.00 ± 0.11	0.510	0.173*
PE(P-18:0/0:0)	0.34 ± 0.02†	0.39 ± 0.02	0.520	0.100
PE(P-20:0/0:0)	0.32 ± 0.02†	0.37 ± 0.02	0.549	0.026
SM(d18:0/14:0)	0.98 ± 0.04§	1.17 ± 0.04	0.601	0.144*
SM(d18:0/18:0)	2.55 ± 0.19‡	3.03 ± 0.19	0.662**	-0.087
SM(d18:0/22:0)	1.25 ± 0.09§	1.70 ± 0.09	0.747***	-0.090
SM(d18:1/24:1) + SM(d18:2/24:0)	1.42 ± 0.03†	1.33 ± 0.04	0.351	0.305**
SM(42:3)	1.45 ± 0.04§	1.27 ± 0.04	0.320	0.368**
SM(38:0)	1.18 ± 0.07§	1.58 ± 0.08	0.759***	-0.111
TG(42:0)	0.27 ± 0.11§	0.31 ± 0.04	0.674**	-0.084
TG(44:0)	0.39 ± 0.12§	0.57 ± 0.06	0.707***	-0.121
TG(44:1)	0.41 ± 0.17§	0.53 ± 0.06	0.674**	-0.095
TG(45:0)	0.69 ± 0.06‡	0.78 ± 0.04	0.611	-0.059
TG(46:0)	0.50 ± 0.10§	0.86 ± 0.09	0.738***	-0.150*
TG(46:1)	0.46 ± 0.11§	0.78 ± 0.08	0.702***	-0.141*
TG(46:2)	0.45 ± 0.12§	0.68 ± 0.07	0.670**	-0.125
TG(47:0)	0.74 ± 0.07§	1.10 ± 0.10	0.698***	-0.133
TG(47:1)	0.66 ± 0.08§	0.94 ± 0.08	0.668**	-0.121
TG(47:2)	0.59 ± 0.07§	0.76 ± 0.06	0.625*	-0.098
TG(48:0)	0.77 ± 0.10§	1.35 ± 0.13	0.736***	-0.176*
TG(52:4)	1.90 ± 0.15§	2.91 ± 0.29	0.686***	-0.194**
TG(48:1)	0.72 ± 0.10§	1.23 ± 0.10	0.714***	-0.170*
TG(55:2)	0.90 ± 0.07§	1.28 ± 0.09	0.676**	-0.112
TG(55:3)	1.11 ± 0.07§	1.50 ± 0.11	0.628*	-0.082
TG(55:4)	1.24 ± 0.08†	1.52 ± 0.10	0.567	-0.064
TG(58:5)	2.84 ± 0.23§	4.14 ± 0.36	0.655**	-0.125
TG(48:2)	0.63 ± 0.08§	1.00 ± 0.08	0.675**	-0.141*
TG(48:3)	0.56 ± 0.08§	0.84 ± 0.08	0.639*	-0.139
TG(49:0)	0.76 ± 0.09§	1.28 ± 0.14	0.710***	-0.157*
TG(49:1)	0.94 ± 0.10§	1.55 ± 0.15	0.678**	-0.149*
TG(49:2)	0.87 ± 0.08§	1.30 ± 0.10	0.656**	-0.128
TG(50:0)	0.78 ± 0.14§	1.50 ± 0.17	0.738***	-0.172*

TG(50:1)	1.14 ± 0.07§	1.63 ± 0.09	0.714***	-0.185**
TG(50:2)	1.17 ± 0.07§	1.61 ± 0.09	0.688***	-0.159*
TG(50:3)	1.04 ± 0.06§	1.39 ± 0.08	0.638*	-0.116
TG(50:4)	0.93 ± 0.08‡	1.24 ± 0.11	0.588	-0.125
TG(51:1)	1.04 ± 0.11§	1.78 ± 0.17	0.697***	-0.166*
TG(51:3)	1.29 ± 0.10§	1.87 ± 0.14	0.646*	-0.132
TG(51:2)	1.11 ± 0.06†	1.44 ± 0.10	0.575	-0.104
TG(52:0)	0.63 ± 0.18§	1.17 ± 0.17	0.754***	-0.135
TG(52:1)	1.05 ± 0.11§	1.79 ± 0.14	0.737***	-0.209**
TG(52:2)	1.45 ± 0.05‡	1.65 ± 0.06	0.641*	-0.157*
TG(53:0)	1.02 ± 0.03†	1.13 ± 0.04	0.663**	-0.077
TG(53:1)	0.76 ± 0.08§	1.29 ± 0.11	0.721***	-0.173*
TG(53:2)	1.37 ± 0.10§	1.91 ± 0.15	0.646*	-0.135
TG(53:3)	1.35 ± 0.08†	1.70 ± 0.11	0.589	-0.096
TG(53:3)	0.49 ± 0.07§	0.67 ± 0.06	0.700***	-0.113
TG(54:1)	0.74 ± 0.15§	1.37 ± 0.16	0.748***	-0.146*
TG(54:2)	1.35 ± 0.09§	1.90 ± 0.12	0.693***	-0.172*
TG(54:5)	3.24 ± 0.16†	3.94 ± 0.24	0.625*	-0.153*
TG(56:1)	0.55 ± 0.08§	0.72 ± 0.06	0.694***	-0.103
TG(56:2)	1.01 ± 0.15§	1.45 ± 0.13	0.695***	-0.114
TG(56:3)	1.47 ± 0.11‡	1.83 ± 0.12	0.622*	-0.059
TG(56:7)	14.47 ± 1.20†	19.70 ± 1.96	0.546	-0.047
TG(58:1)	0.60 ± 0.08‡	0.73 ± 0.06	0.660**	-0.089
TG(58:2)	0.73 ± 0.08‡	0.90 ± 0.07	0.665**	-0.098
TG(58:3)	1.06 ± 0.11†	1.30 ± 0.10	0.648**	-0.111
TG(60:2)	0.60 ± 0.06‡	0.73 ± 0.06	0.650**	-0.061
TG(60:3)	0.85 ± 0.08†	0.97 ± 0.07	0.633*	-0.047

Values depicted as mean ± SEM; relative quantifications. Applied in validation cohort; n = 91, NAFL; n = 90, NASH. AC, acylcarnitine; Cer, ceramide; ChoE, cholesteryl ester; Di-HOME, dihydroxy-octadecadenoic acid; DG, diglyceride; LPE, lysophosphatidylethanolamine; LPI, lysophosphatidylinositol; PC, phosphatidylcholine; PE, phosphatidylethanolamine; SM, sphingomyelin; TG, triglyceride. * $p < 0.05$; ** $p < 0.01$; *** $p < 0.001$. NAFL *versus* NASH: $^{\dagger}p < 0.05$; $^{\ddagger}p < 0.01$; $^{\S}p < 0.001$.

Table S3. Specific serum lipid signature of advanced liver fibrosis and its association with IGF-1 serum levels.

	Serum lipidomics		Correlation with serum IGF-1
	F0-2	F3-4	
Cholic acid	0.52 ± 0.11	0.97 ± 0.37†	0.188*
Deoxycholic acid	0.29 ± 0.03	0.42 ± 0.08†	0.084
Taurocholic acid	0.41 ± 0.10	8.70 ± 7.43‡	-0.116
Taurodeoxycholic acid	0.19 ± 0.04	1.23 ± 0.58‡	-0.067
Taurochenodeoxycholic acid	0.33 ± 0.06	5.95 ± 5.12‡	-0.120
Tauroursodeoxycholic + Taurohyodeoxycholic acid	0.40 ± 0.05	2.40 ± 1.82†	-0.136
Glycocholic acid	0.26 ± 0.08	2.18 ± 1.47‡	-0.073
Glycochenodeoxycholic acid	0.21 ± 0.02	1.06 ± 0.65‡	-0.110
Glycodeoxycholic acid	0.15 ± 0.02	0.62 ± 0.25‡	-0.038
ChoE(18:2)	0.72 ± 0.02	0.61 ± 0.06†	0.085
PC(18:0/22:4)	0.99 ± 0.04	1.14 ± 0.10†	0.097
PC(17:1/18:1)	0.75 ± 0.02	0.59 ± 0.08†	0.070
PC(17:0/18:2)	0.73 ± 0.02	0.59 ± 0.05†	0.159*
PC(15:0/22:6)	2.21 ± 0.09	1.39 ± 0.21‡	0.113
FFA 20:3n-x	0.49 ± 0.01	0.64 ± 0.08†	0.003
Mead acid (20:3n-9)	1.06 ± 0.04	1.97 ± 0.47§	-0.018
Adrenic acid (22:4n-6)	1.27 ± 0.04	1.72 ± 0.18†	-0.016
Docosapentaenoic acid (22:5n-6)	0.82 ± 0.03	1.06 ± 0.10‡	0.013
PC(18:1/0:0)	0.09 ± 0.00	0.06 ± 0.01‡	0.012
PC(20:2/0:0)	1.78 ± 0.08	2.73 ± 0.47‡	0.012
PC(20:3/0:0)	1.74 ± 0.07	2.24 ± 0.24†	0.018
PE(P-20:2/0:0)	0.46 ± 0.02	0.35 ± 0.06†	-0.007
SM(33:1)	1.06 ± 0.02	0.87 ± 0.08†	0.082
SM(d18:1/17:0)	1.16 ± 0.02	0.98 ± 0.08†	0.014
SM(d18:2/14:0)	1.20 ± 0.03	0.93 ± 0.12†	-0.056
TG(58:5)	3.39 ± 0.23	3.95 ± 0.52†	0.120

Values depicted as mean ± SEM; relative quantifications. Applied in validation cohort; n ≤ 174, F0-2; n ≤ 15, F3-4. ChoE, cholesteryl ester; FFA, free fatty acid; PC, phosphatidylcholine; PE, phosphatidylethanolamine; SM, sphingomyelin; TG, triglyceride. *$p < 0.05$. F0-2 *versus* F3-4: †$p < 0.05$; ‡$p < 0.01$; §$p < 0.001$.

2 Supplementary Figures

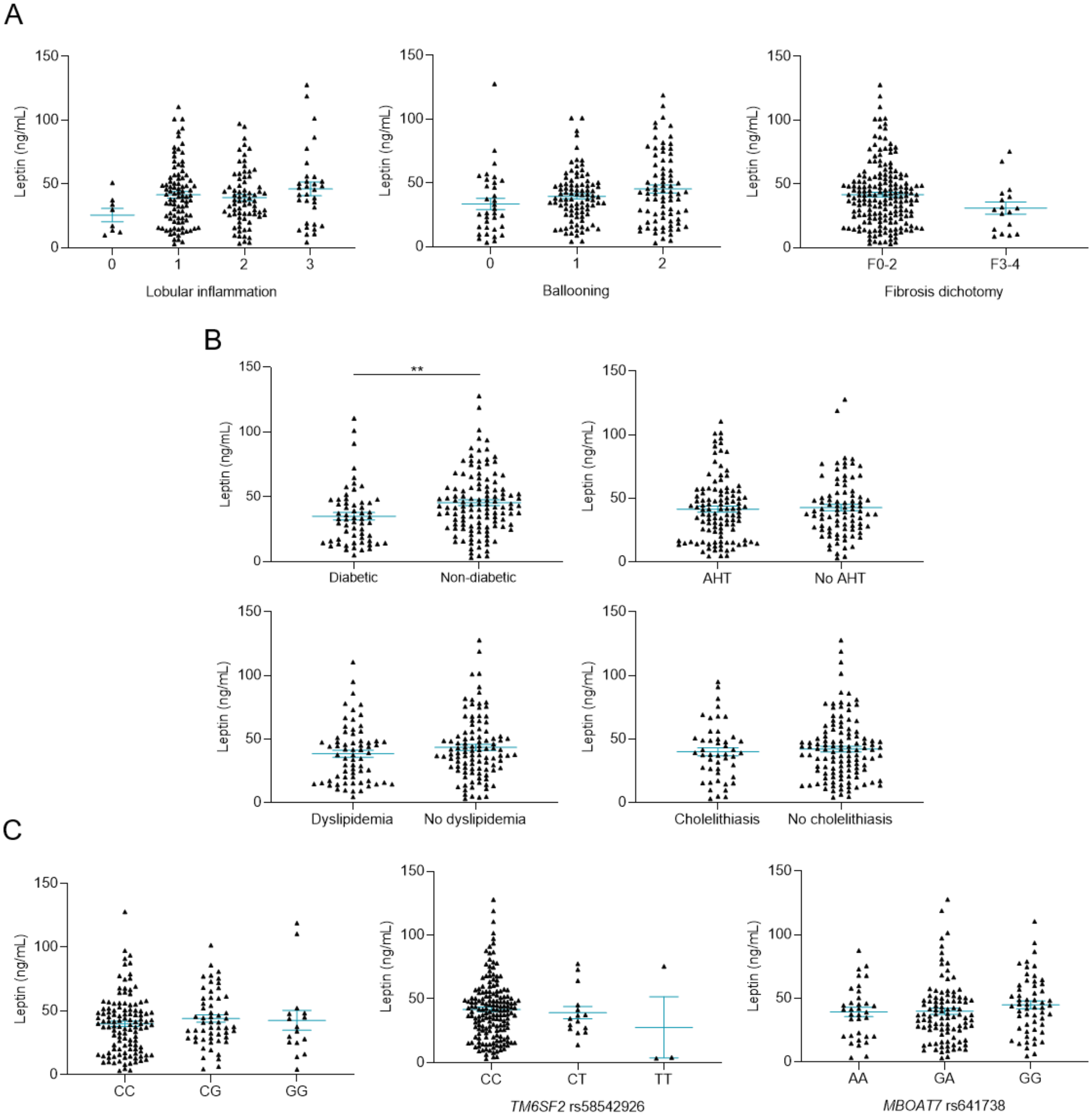

Figure S1. Leptin levels in the validation cohort. No significant differences were found in circulating leptin levels according to (A) severity of liver histology: lobular inflammation (n = 8, score 0; n = 92, score 1; n = 73, score 2; n = 32, score 3), ballooning (n = 34, score 0; n = 92, score 1; n = 79, score 2), fibrosis dichotomy (n = 184, F0-2; n = 17, F3-4); (B) presence of co-morbidities: diabetes (n = 60, diabetic; n = 121, non-diabetic), AHT (n = 110, AHT; n = 88, no AHT), dyslipidemia (n = 67, dyslipidemia; n = 104, no dyslipidemia), cholelithiasis (n = 47, cholelithiasis; n = 118, no cholelithiasis); and (C) presence of NAFLD-risk conferring polymorphisms: PNPLA3 rs738409 (n = 117, CC; n = 55, CG; n = 16, GG), TM6SF2 rs58542926 (n = 170, CC; n = 15, CT; n = 3, TT), MBOAT7 rs641738 (n = 34, AA; n = 98, GA; n = 56, GG). ** $p < 0.01$.

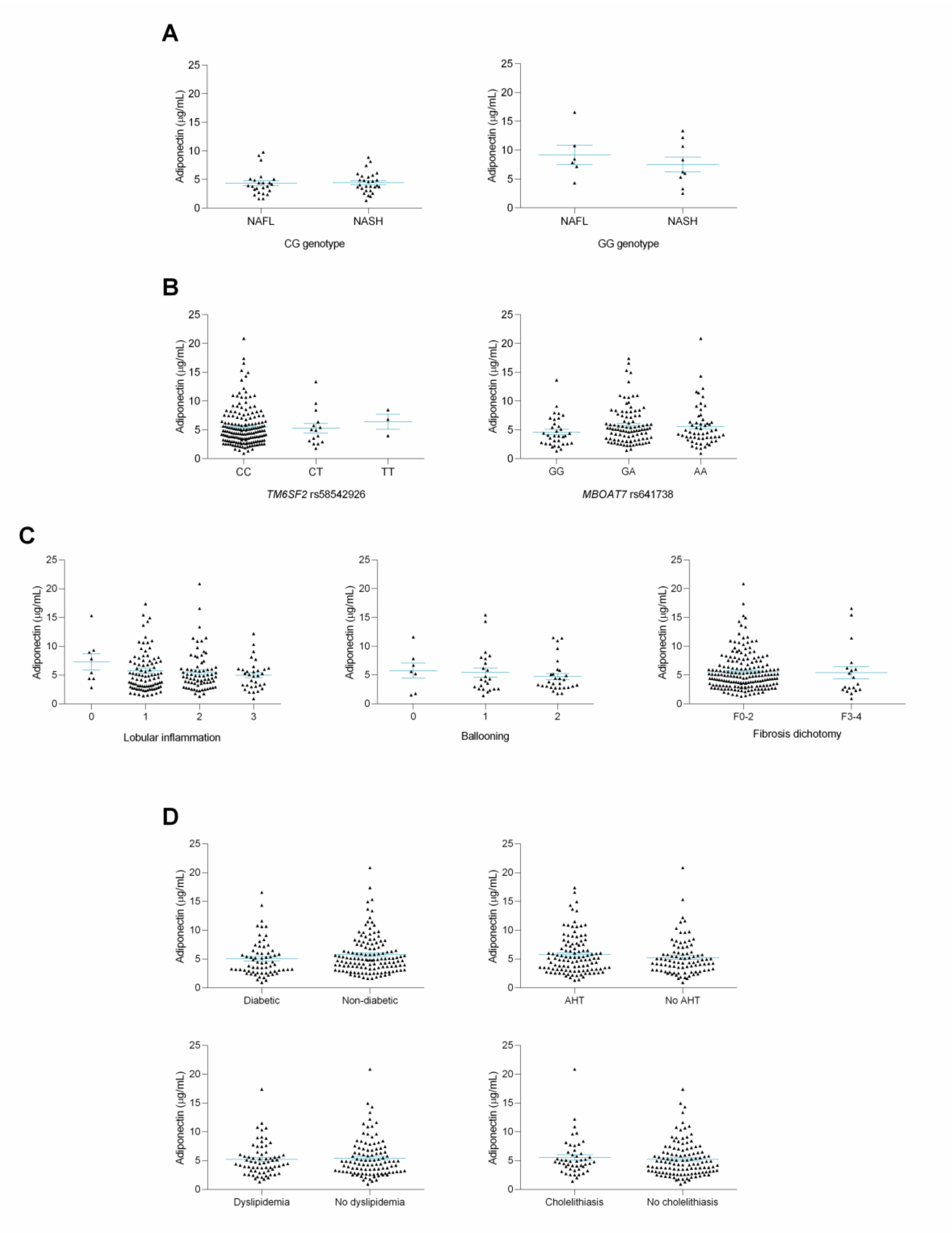

Figure S2. Adiponectin levels in the validation cohort. No significant differences were found in circulating adiponectin levels according to (**A**) *PNPLA3* rs738409 CG genotype (n = 24, NAFL; n = 28, NASH) or GG genotype (n = 6, NAFL; n = 9, NASH); (**B**) *TM6SF2* rs58542926 (n = 170, CC; n = 15, CT; n = 3, TT) and *MBOAT7* rs641738 (n = 34, AA; n = 98, GA; n = 56, GG); (**C**) severity of liver histology: lobular inflammation (n = 8, score 0; n = 92, score 1; n = 73, score 2; n = 32, score 3), ballooning (n = 7, score 0; n = 23, score 1; n = 29, score 2), fibrosis dichotomy (n = 183, F0-2; n = 19, F3-4); and (**D**) presence of co-morbidities: diabetes (n = 66, diabetic; n = 132, non-diabetic), AHT (n

= 110, AHT; n = 88, no AHT), dyslipidemia (n = 67, dyslipidemia; n = 104, no dyslipidemia) and cholelithiasis (n = 47, cholelithiasis; n = 118, no cholelithiasis).

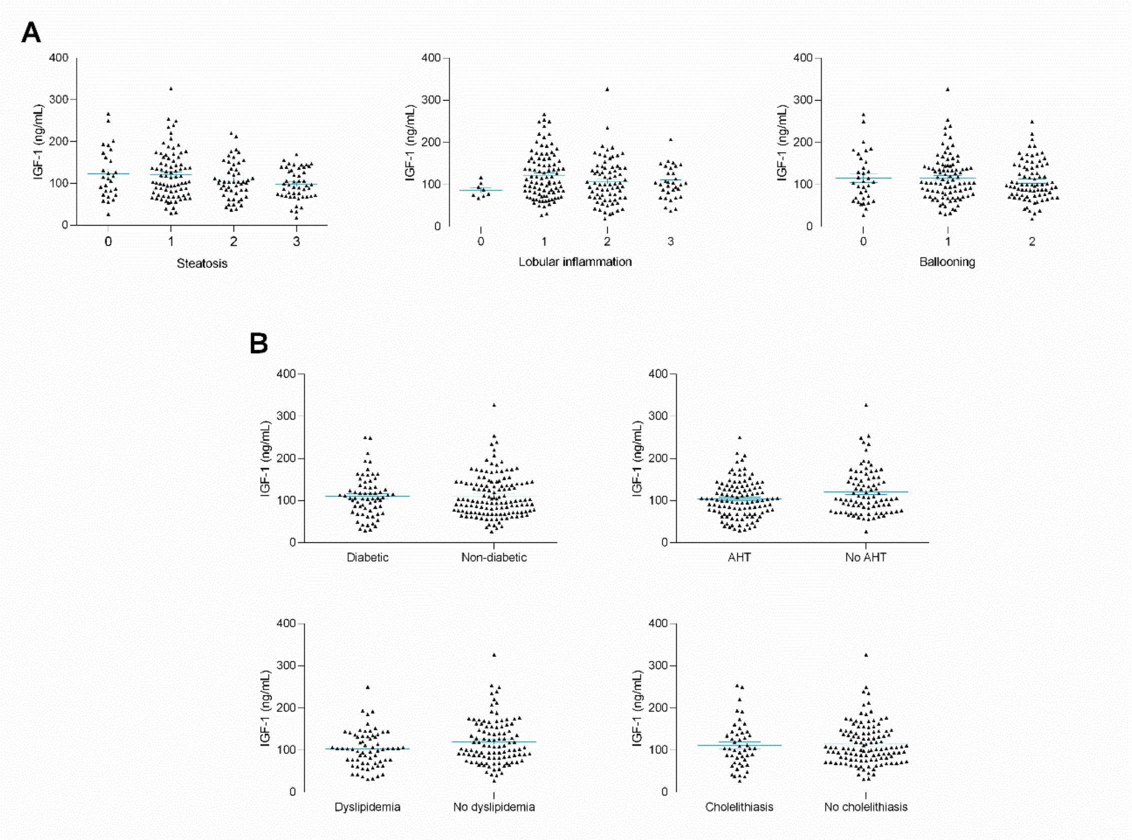

Figure S3. IGF-1 levels in the validation cohort. No significant differences were found in circulating levels of IGF-1 according to (**A**) severity of liver histology: steatosis (n = 28, score 0; n = 80, score 1; n = 50, score 2; n = 48, score 3), lobullar inflammation (n = 8, score 0; n = 92, score 1; n = 73, score 2; n = 32, score 3), balloning (n = 34, score 0; n = 92, score 1; n = 79, score 2); and (**B**) presense of co-morbidities: diabetes (n = 132, diabetic; n = 66, non-diabetic), AHT (n = 110, AHT; n = 88, no AHT), dyslipidemia (n = 67, dyslipidemia; n = 104, no dyslipidemia) and cholelithiasis (n = 47, cholelithiasis; n = 118, no cholelithiasis).

3 Supplementary References

1. Juluri R, Vuppalanchi R, Olson J, Ünalp A, Natta ML Van, Cummings OW, Tonascia J, Chalasani N. Generalizability of the NASH CRN Histological Scoring System for Nonalcoholic Fatty Liver Disease. *J Clin Gastroenterol* (2011) **45**:55–58. doi:10.1097/MCG.0b013e3181dd1348
